# Probiotic supplementation promotes a reduction in T‐cell activation, an increase in Th17 frequencies, and a recovery of intestinal epithelium integrity and mitochondrial morphology in ART‐treated HIV‐1‐positive patients

**DOI:** 10.1002/iid3.160

**Published:** 2017-04-20

**Authors:** Gabriella d'Ettorre, Giacomo Rossi, Carolina Scagnolari, Mauro Andreotti, Noemi Giustini, Sara Serafino, Ivan Schietroma, Giuseppe Corano Scheri, Saeid Najafi Fard, Vito Trinchieri, Paola Mastromarino, Carla Selvaggi, Silvia Scarpona, Gianfranco Fanello, Fausto Fiocca, Giancarlo Ceccarelli, Guido Antonelli, Jason M. Brenchley, Vincenzo Vullo

**Affiliations:** ^1^ Department of Public Health and Infectious Diseases Azienda Policlinico Umberto I of Rome Rome Italy; ^2^ School of Biosciences Veterinary Medicine University of Camerino Matelica Italy; ^3^ Laboratory Affiliated to Istituto Pasteur Italia—Fondazione Cenci Bolognetti Department of Molecular Medicine Sapienza University of Rome Rome Italy; ^4^ Department of Therapeutic Research and Medicines Evaluation Italian Institute of Health Rome Italy; ^5^ Department of Public Health and Infectious Diseases Sapienza University of Rome Rome Italy; ^6^ Section of Microbiology Department of Public Health and Infectious Diseases Sapienza University of Rome Rome Italy; ^7^ Department of Emergency Surgery—Emergency Endoscopic Unit Policlinico Umberto I Sapienza University of Rome Rome Italy; ^8^ Laboratory of Parasitic Diseases National Institute of Allergy and Infectious Diseases, NIH Bethesda Maryland USA

**Keywords:** apoptosis, GALT, GUT, HIV‐1, HSP60, IELs, immunity, probiotics, T‐cell activation, Tc1, Tc17, Th1, Th17

## Abstract

**Introduction:**

HIV infection is characterized by a persistent immune activation associated to a compromised gut barrier immunity and alterations in the profile of the fecal flora linked with the progression of inflammatory symptoms. The effects of high concentration multistrain probiotic (Vivomixx®, Viale del Policlinico 155, Rome, Italy in EU; Visbiome®, Dupont, Madison, Wisconsin in USA) on several aspects of intestinal immunity in ART‐experienced HIV‐1 patients was evaluated.

**Methods:**

A sub‐study of a longitudinal pilot study was performed in HIV‐1 patients who received the probiotic supplement twice a day for 6 months (T6). T‐cell activation and CD4+ and CD8+ T‐cell subsets expressing IFNγ (Th1, Tc1) or IL‐17A (Th17, Tc17) were stained by cytoflorimetric analysis. Histological and immunohistochemical analyses were performed on intestinal biopsies while enterocytes apoptosis index was determined by TUNEL assay.

**Results:**

A reduction in the frequencies of CD4^+^ and CD8^+^ T‐cell subsets, expressing CD38^+^, HLA‐DR^+^, or both, and an increase in the percentage of Th17 cell subsets, especially those with central or effector memory phenotype, was recorded in the peripheral blood and in gut‐associated lymphoid tissue (GALT) after probiotic intervention. Conversely, Tc1 and Tc17 levels remained substantially unchanged at T6, while Th1 cell subsets increase in the GALT. Probiotic supplementation was also associated to a recovery of the integrity of the gut epithelial barrier, a reduction of both intraepithelial lymphocytes density and enterocyte apoptosis and, an improvement of mitochondrial morphology sustained in part by a modulation of heat shock protein 60.

**Conclusions:**

These findings highlight the potential beneficial effects of probiotic supplementation for the reconstitution of physical and immunological integrity of the mucosal intestinal barrier in ART‐treated HIV‐1‐positive patients.

## Introduction

Damage to the intestinal epithelium with enterocyte apoptosis and compromising tight junction complexes, massive depletion of CD4^+^ T‐cells in gastrointestinal (GI) tract, and preferential decrease of Th17 cells from GI tract contributes significantly to translocation of commensal microbial products from gut into circulation which is considered a major driver of chronic immune activation in HIV‐1‐infected humans and in the experimental model SIV‐infected rhesus macaques (RMs) 1, 2, 3_._ In chronically SIV‐infected RMs, microbial translocation occurs during the late stage of acute SIV infection and is associated with epithelial damage of the GI tract and an inability of macrophages in intestinal lamina propria to phagocytose translocated microbial constituents 4. Conversely, in the chronic phase of SIV infection in sooty mangabeys, no evidence of breakdown in intestinal epithelial barrier, no enhancement of bacterial translocation, and no chronic immune activation are observed 4, 5, 6. Given the strong relationship between the impairment of GI tract and the presence of various alterations in gut microbiota (dysbiosis) during chronic HIV‐1 infection 7, 8, 9, 10, the development of adjunctive strategies aimed at recovering the intestinal homeostasis could benefit HIV‐1‐infected individuals, particularly those on antiretroviral therapies. In this regard, the administration of oral probiotic alone or in combination with interleukin‐(IL) 21 promotes increased reconstitution of intestinal CD4^+^ T‐cell during ART supplementation in SIV‐infected macaques 11; interestingly, the effects on Th17 cell subset frequency were different depending on whether the probiotic were taken alone or in combination with IL‐21 11, 12. Macaques treated with a *Lactobacillus*‐containing formulation showed also a decreased IDO1 activity improving the preservation of Th17 cells during pathogenic SIV infection 13. Most of our understanding on potential benefits imparted to HIV‐1‐positive individuals by dietary supplementation with probiotics are derived from animal models of lentiviral disease or from the evaluation of the systemic effects of probiotic 11, 12, 13, 14, 15, 16, 17 and only few studies have focused analyses on gut microbiota 18, 19, 20, 21, 22, 23, gastrointestinal anatomy, and physiology 21 and potential benefits of probiotic administration in HIV‐1‐infected patients. Although ART subjects might benefit from probiotic supplementation, these individuals might also be at risk for unexpected adverse reactions such as sepsis and the limited understanding about the mechanisms of probiotics action is a major drawback for the prediction of probiotic safety 24. Herein, we decided to carry on a pilot study to assess the safety and efficacy of a probiotic supplementation selecting for the supplementation to ART patients a high concentration multi strain probiotic mixture (Vivomixx® in Europe; Visbiome ® in USA) which has been extensively used in diseases characterized by mucosal ulceration and exposure of the submucosa, that is, inflammatory bowel diseases 25, 26. The aim of the study was also to verify whether this preparation could play a role in restoring GI tract immunity and intestinal barrier integrity with consequent reduction of inflammation and enhancement of the recovery of CD4^+^ T cells and, in particular, Th17 cells. We observed that this dietary management is associated with increased frequencies of mucosal and systemic Th17 cells and a strong decrease in T‐cell activation. Furthermore, we provide, for the first time to our knowledge, direct in vivo evidence that this specific probiotic supplementation caused profound histomorphological tissue changes in the intestinal epithelium of HIV‐1‐infected patients and identify the recovery of the integrity of the gut epithelial barrier strictly dependent to a reduction of both intraepithelial lymphocytes (IELs) density and enterocyte apoptosis and an improvement of mitochondrial morphology sustained in part by a modulation of heat shock protein 60 (HSP60) protein expression. These findings underscore the benefits and safety of this specific formulation as adjunctive and practical intervention for the reconstitution of physical and immunological integrity of the mucosal intestinal barrier and reducing systemic T‐cell immune activation in ART‐treated HIV‐1‐positive patients, at least in this pilot study.

## Materials and Methods

### Study design, recruitment, and study eligibility criteria

This is a sub‐study of a pilot longitudinal, non‐randomized designed, single arm study (clinicaltrials.gov registry [number NCT02276326]; Consort Checklist [Supplementary Fig. S1], and Flow Diagram [Supplementary Fig. S2]) including 10 HIV‐1‐positive patients because we had to be sure about the safety of the tested probiotic.

Patients were selected for the present study, if they met the following inclusion criteria: (i) who had signed the informed consent; (ii) men or women at least 18 years of age; (iii) in ART; and (iv) with HIV‐1 RNA <37 copies/mL and CD4^+^ T counts >400 cells/mm^3^. Exclusion criteria included: (i) known or suspected allergy or intolerance to the specific probiotic formulation; (ii) use of probiotics or antibiotics during the 3 weeks prior the enrollment; (iii) drug addiction; (iv) history of or current inflammatory diseases of the small or large intestine; (v) diarrhea; (vi) any current, past, or systemic malignancy; and (vii) pregnancy.

From May 2014 to February 2015, 10 HIV‐1‐positive patients successfully ART‐treated were recruited at the Division of Infectious Diseases, Department of Public Health and Infectious Diseases, Hospital of Sapienza University of Rome (Italy).

Patients received a high concentration lyophilized multistrain probiotic supplement (*Lactobacillus plantarum* DSM24730, *Streptococcus thermophilus* DSM24731, *Bifidobacterium breve* DSM24732, *L. paracasei* DSM24733, *L. delbrueckii* subsp. *bulgaricus* DSM24734, *L. acidophilus* DSM 24735, *B. longum* DSM24736, *B. infantis* DSM24737) twice a day for 6 months. This formulation is now commercialized as Vivomixx®; Visbiome® 27. The probiotic preparation was administered per os at a daily dosage of 1.8 × 10^12^ live bacteria.

The study was approved by the institutional review board (Department of Public Health and Infectious Diseases, Sapienza University of Rome) and the Ethics Committee (Sapienza University of Rome) on 16 January 2014. All study participants signed written informed consent. No adverse event was observed during the follow‐up and all subjects maintained undetectable plasmatic viral load before and after probiotic assumption (HIV‐1 RNA [< 37 copies/mL]).

### Laboratory procedures

All patients underwent endoscopic procedures and blood collection prior to initiation of probiotic supplementation (T0) and after 6 months (T6). Colonic washing was carried out by PEG administration 24 h before the examination. The endoscopic procedure was performed with conscious sedation (midazolam 5 mg/iv) using large cup forceps (Radial Jaw 4, Boston Scientific, Natick, MA). All individuals underwent a total colonoscopy and retrograde ileoscopy for at least 10 cm of distal ileum with conventional or slim scope (model CF or PCF‐160 AI, Olympus Medical Europe GmbH, Hamburg, Germany). Specimens (two biopsies from each site) from the terminal ileum, cecum, ascending, transverse, and descending colon were obtained. Additionally, biopsies for histology and immunohistochemistry evaluations were collected. Lastly, fecal samples were collected before and after 2 and 6 months of probiotic supplementation. T‐cell phenotype and activation markers were analyzed on freshly isolated peripheral blood mononuclear cells (PBMCs) and lamina propria lymphocytes (LPLs). T‐cells subpopulation and cytokine expression were evaluated after overnight‐cell culture.

### Specimen processing

Twenty milliliters of whole blood were collected by venipucture in Vacutainer tubes containing ethylenediaminetetraacetic acid (BD Biosciences, San Jose, CA) at each study visit. Plasma was immediately separated by centrifugation and stored at −80°C for further analysis.

PBMCs were separated on Ficoll gradient centrifugation (Lympholyte, Cedarlane Labs, Hornby, Ontario, Canada), and washed twice in phosphate‐buffered saline. Freshly isolated PBMCs were used immediately for immune phenotyping and activation staining or overnight cultured for stimulation. Biopsies from intestinal sites were mixed with each other and processed. Briefly, biopsies collected in RPMI 1640 (heat inactivated 10% fetal bovine serum) were washed twice with EDTA wash media, resuspended, and incubated for 1 h at room temperature in EDTA solution 5 mM on automatic shaker. Supernatant containing intraepithelial lymphocytes was removed, and biopsies were digested by 1‐h incubation at 37°C in pre‐warmed RPMI 1640 (heat inactivated 10% fetal bovine serum) with 1 mg/mL collagenase (Sigma–Aldrich, Milan, Italy) and 1.5 U DNAse I (Sigma–Aldrich), bringing to the isolation of LPLs, then filtered through a 70 µm cell strainer (Becton Dickinson).

### Cell cultures

PBMCs and LPLs were seeded at concentrations of 2 × 10^6^ cells/mL and 1.5 × 10^6^ cells/mL, respectively, with RPMI media plus 20% heat inactivated fetal bovine serum (FBS) and incubated overnight at 37°C and 5% CO_2_ in the presence of medium alone or supplemented with phorbol myristyl acetate (PMA) (3 ng/mL, Sigma–Aldrich) and ionomycin (1 µg/mL, Sigma–Aldrich). BD GolgiStop (Becton Dickinson) was added to all culture conditions. Cells were then collected, washed, permeabilized, and stained for T‐cell phenotype and cytokine expression.

### Monoclonal antibody and T‐cell phenotyping

Phenotype and activation were evaluated by 7‐color flow cytometric analysis on freshly isolated PBMCs and LPLs using the following anti‐human monoclonal antibodies: CD3‐PerCP, CD4‐APC‐Vio770, CD8‐FITC, CD45RO‐PEVio770, CD27‐VioBlue, CD38‐APC, and HLA‐DR‐PE (Miltenyi Biotec, Bergisch Gladbach, Germany). Gating strategy for flow cytometry analysis of peripheral blood and GALT is provided in Supplementary Figure S3. T‐cell subpopulations were identified according to the following phenotypic combinations: CD27^+^CD45RO^+^ (Central Memory) and CD27^−^CD45RO^+^CD4^+^ (Effector Memory) cells. Cultured cells were fixed, permeabilized (BD Cytofix/Cytoperm, Becton Dickinson), and stained with combinations of surface and intracellular fluorochrome‐labeled monoclonal antibodies: CD3‐PerCP, CD4‐APC‐Vio770, CD8‐FITC, CD45RO‐PE‐Vio770, CD27‐VioBlue, IFNγ‐APC, and IL‐17A‐PE (Miltenyi Biotec).

CD3^+^CD4^+^ cells expressing IFNγ or IL‐17A were identified as Th1 and Th17, respectively; CD3^+^CD8^+^ cells expressing IFNγ or IL‐17A were identified as Tc1 and Tc17, respectively.

Acquisitions were performed on Miltenyi Biotec flow cytometer‐MACSQuant Analyzer (eight fluorescence channels, three lasers). Gating analysis and data were analyzed using MACSQuantify software 2.5 (Miltenyi Biotec). The same gating strategy was applied to all samples. At least 100,000 and 10,000 events in the CD3+ lymphocytes gate were analyzed for PBMCs and LPLs, respectively. Isotype controls were used as negative controls to differentiate non‐specific background signal from specific antibody signal of CD38, HLA‐DR, IL‐17A, and IFNγ markers.

### Real‐time PCR assay for bifidobacteria

Bacterial DNA from fecal samples collected before and after 2 and 6 months of probiotics supplementation was extracted using the QIAamp DNA Stool Mini Kit (Qiagen, Hilden, Germany) as previously reported 28. Then, real‐time PCR was performed evaluate bifidobacteria levels as previously described 28, 29. Briefly, PCR amplification and detection were performed on optical‐grade 96‐well plates using the Applied Biosystems 7500 Real‐Time PCR instrument (Applied Biosystems, Inc., Norwalk, Conn). The reaction mixture (25 μL) was composed of SensiMix SYBR Low‐ROX (BIOLINE, Taunton, MA), 500 nM primers for *Bifidobacterium* genus, and 2.5 μL of template DNA. A melting curve analysis was made after amplification to distinguish target amplicons from aspecific non‐target PCR products. Standard curves were made by using 10‐fold dilutions of DNA extracted from *Bifidobacterium breve*. All samples were analyzed in duplicate in two independent real‐time PCR assays.

### Virological analysis

HIV‐1 RNA copy number was measured in plasma by using Versant kPCR (Siemens Healthcare Diagnostic, Inc., Tarrytown, NY). The detection limit was 37 HIV‐1 RNA copies/mL.

### Histological analysis

Histological score analysis of HIV‐1‐positive patients was performed at T0 and T6 to grade: (i) the amount of immune cells infiltration; (ii) the amount of intra‐epithelial lymphocytes (IELs); and (iii) GALT (Peyer patches) activation. Histological analysis comprehended the estimation of cellular infiltrates and aggregation by scoring the number of macrophages, lymphocytes, plasma cells, and neutrophils at 400× magnification. The inflammatory cells number was measured with a semiquantitative method 30, 31, 32 and results are provided as the mean for the entire specimen. In the presence of strong intensity variation of infiltration in the same specimen, the mean for several areas was determined and the specimen was scored accordingly. Neutrophils and macrophages were classified as absent (score 0) when there were no or fewer than 19 cells per high‐power field (HPF) (at a 400× magnification), mild (score 1) for 20–49 cells per HPF, moderate (score 2) for 50–99 cells per HPF, marked or severe (score 3) for 100–200 cells or more per HPF. Histological criteria for normal intestinal features included detection of only a few mononuclear cells in transcytosis across epithelium per HPF, as IELs and no, or only a few scattered neutrophils in the axis of villi and/or between the crypts without tissue changes (no interstitial thickening or aggregates of lymphocytic infiltrates, and lumen free from exudate). The number of inflammatory cells and of lymphoid aggregates was assessed at ×400 and ×100 magnification, respectively, and was scored as described by Dixon et al. 33. For GALT activation, the score was assessed at ×100 magnification, by calculating the total areas of GALT follicles evaluated inside each intestinal section, and subdividing the mean as follow: mean area of GALT follicle extension; score 0, none; score 1, up to 0.008 mm^2^; score 2, up to 0.042 mm^2^; score 3, up to 0.4 mm^2^.

### Immunohistochemical evaluation

Tissues were fixed in 10% neutral buffered formalin (Sigma, Milan, Italy) for paraffin embedding. Paraffin‐embedded sections were cut at 3 μm, deparaffinized, rehydrate, and incubated with the follow primary antibody: HSP60 (mouse anti‐human HSP60/Heat Shock Protein 60 Mab–2E1/53–ThermoFisher,, used at 1:50 dilution). Subsequently, rehydrated sections were treated for endogenous peroxidases neutralization with 3% hydrogen peroxide for 5 min followed by rinsing for 5 min in distilled water. Antigen retrieval was achieved by incubating slides in citrate buffer (pH 6.0) according to antibody datasheet, in a steamer (Black & Decker, Towson, MD) for 20 min. Non‐specific immunoglobulin binding was blocked by incubation of slides with a nonspecific staining protein‐blocking reagent (DakoCytomation, Carpinteria, CA) for 20 min, before application of the primary antibody, used at the optimal dilutions, overnight at 4°C. After the incubation with biotinylated secondary antibodies (goat anti‐mouse IgG–AO433; DAKO, used diluted 1:200), the resultant immune complexes were visualized by the ABC Elite kit (Vector Laboratories, Burlingame, CA) and peroxidase substrate 3,3′‐diaminobenzidine kit (Vector Laboratories) according to the manufacturer's instructions. Then, the slides were counterstained with hematoxylin, dehydrated, and cover slipped. For scoring antigens‐positive cells, stained cells were quantified in different intestinal compartments (villus, apical crypt area, basal crypt area). All cellular types were calculated using a light microscope (Carl Zeiss), at 40× objective, a 10× eyepiece, and a square eyepiece reticule (10 × 10 squares, with a total area of 62,500 µm^2^). Five sites were chosen for each compartment and arithmetic means were calculated for each intestinal region. Results were expressed as immunohistochemistry‐positive cells per 62,500 µm^2^.

### TUNEL for apoptosis evaluation

For the in situ detection of epithelial and lymphocytes apoptotis, terminal deoxynucleotidyl transferase‐mediated digoxigenindeoxyuridine triphosphate nick‐end labeling (TUNEL) was performed on histological sections of endoscopic ileal and colonic biopsy specimens collected from all HIV‐1‐positive patients, 34, 35. The sections were digested with proteinase K treatment, 20 µg/mL (Sigma Chemical, St Louis, MO) for 15 min at RT. After digestion, the sections were washed in tap water. Further, endogenous peroxidase activity was quenched with 3% hydrogen peroxidase for 20 min and then washed in PBS. After equilibration, the sections were incubated at 37°C in a humidified chamber for 1 h in terminal deoxynucleotidyl transferase (TdT) enzyme and then placed in stop/wash buffer for 30 mins. Next, the sections were rinsed in PBS and, thereafter, treated with antidigoxigenin peroxidase for 30 mins in a humidified chamber. Color on apoptotic nuclei was developed using diaminobenzidine and hydrogen peroxide for 1–3 min. Finally, sections were counterstained with Harris hematoxylin. As a positive control, an ApopTag control slide was used, whereas negative control was prepared by omitting the TdT enzyme from the nucleotide mix on seriate sections. Counts were assessed, at a ×200 magnification, by computer‐assisted analysis and the results were expressed as median percentages of positive enterocytes or lymphoid cells inside epithelia or GALT follicles.

### Transmission electron microscopy and immunogold labeling

A small fragment of each intestinal section was cut into small pieces (<1 mm^3^) and fixed for 60 min at 4°C in periodate‐lysine‐paraformaldehyde fixative (PLP) 36. Pieces of tissue were washed three times in 50 mM Sorensen's phosphate buffer, 200 mM sucrose, and 100 mM lysine monochlorhydrate for 20 min,at 4°C, dehydrated through a series of ethanol washes (50%, 70%, 95%, 100%), and infiltrated with 1:1 pure ethanol: London Resin (LR) White for 60 min, followed by pure LRWhite, three times for 60 min at 4°C. Next, the specimens were embedded in gelatine capsules filled with LRWhite and polymerized at 50°C for 48 h. All of the ultrathin sections (<80 nm) were microtomed with an RMC MTX ultra microtome (Elexience), placed on 200 mesh nickel grids coated with polylysine (dilition 1:1000), and stabilized for 1 day at RT. For immunogold labeling, section grids were floated on drops of reactive media. Non‐specific binding sites were coated with 1% BSA and 1% normal goat serum for 60 min at RT. Next, antibody incubation was carried out at 4°C overnight in a wet chamber with mouse anti HSP60 antibody (dilution 1:100). in 1% BSA, 50 mM Tris–HCl, pH 7.4. Tissue sections were successively washed three times in 50 mM Tris–HCl, pH 7.4, and pH 8.2 at RT. They were then incubated in a wet chamber for 45 min at RT in 1% BSA, 50 mM Tris–HCl, pH 8.2, and labeled with 10 nm gold conjugated goat anti‐mouse IgG ([Tebu] dilution 1:25) in 1% BSA, 50 mM Tris–HCl, pH 8.2. After three washing in 50 mM Tris–HCl, pH 8.2 and pH 7.4. and in filtrated distilled water, the sections were then contrasted with aqueous solution of uranyl acetate followed by lead citrate before examining in transmission electron microscope (Jeol 1200EX, Tokyo, Japan) equipped with a MegaViewII digital camera and AnalySIS software (Eloise).

### Statistical analysis

Statistical analyses were performed using SPSS software, version 22.00 (IBM, Somers, NY) on data obtained from peripheral blood, gut, and stool samples of HIV‐1‐positive patients before and after probiotic supplementation. Data obtained at T0 and T6 analysis in peripheral blood and intestinal districts were compared by Wilcoxon test for paired samples. The same test was also used to evaluate data obtained from stool samples at T0, T2, and T6. Results are given as medians, ranges, and percentages. *P* values <0.05 were considered statistically significant. All graphs were generated by using GraphPad Prism (version 5.00).

## Results

### Participant characteristics

All of the study participants were Caucasian men (age [median/range]: 42/22–53 years). They started ART during chronic HIV‐1 infection (baseline CD4^+^ T‐cell count [median/IQR]: 255/42.75–406.75 cells/mm^3^, baseline HIV‐1 RNA copies [median/IQR]: 5.0/4.81–5.61 Log/mL). At study enrolment all HIV‐1‐positive patients received antiretroviral therapy for a median of 6 years (IQR, 1.75–16.25 years); all patients had been virologically suppressed (<37 HIV‐1 RNA copies/mL) for at least 1 year and median CD4^+^ T cell count was 674 cells/mm^3^ (IQR, 564–824 cells/mm^3^).

### Product tolerability, safety, and action

In all study participants, self‐reported adherence to probiotic supplementation was excellent, and no adverse reactions were reported by the patients or identified by the clinicians.

All subjects maintained undetectable plasmatic viral load after probiotic assumption and the median CD4^+^ T‐cell count was 683 cells/mm^3^ (IQR, 610–818 cells/mm^3^).

**Figure 1:**
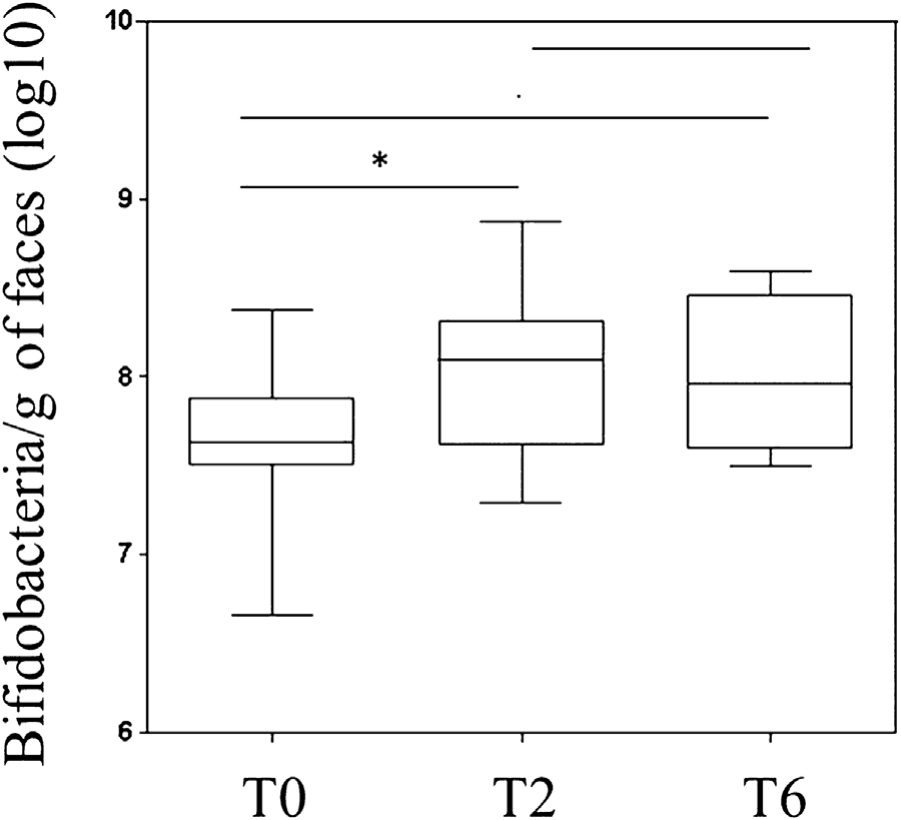
Bifidobacteria in fecal samples of HIV‐infected subjects at enrolment (T0) and after 2 (T2) and 6 months (T6) of probiotic supplementation. Box and whisker plots based on Log 16S rRNA gene copies per gram of stool. The horizontal line in the middle of each box represents the median, while the top and bottom borders of the box represent the 75% and 25% percentiles, respectively. *P < 0.05 (T0 vs. T2); **P < 0.05 (T0 vs. T6), using Wilcoxon test.

To assess whether all HIV‐1‐infected patients had been compliant with the protocol, we measured levels of *Bifidobacteria* spp. in stool samples collected before and after 2 and 6 months of probiotic supplementation We found that fecal *Bifidobacteria* spp. increased significantly in all patients, compared to their basal level, from two months of supplementation (*P* = 0.019) and remained stable throughout the study (Fig. 1) confirming adherence of the patients to the regimen.

### Baseline CD4^+^ and CD8^+^ T‐cells subpopulations

In HIV/SIV‐infected individuals, subtle T‐cell phenotypic differences have been described within small intestinal versus large intestinal T‐cell populations but most important is the dramatic depletion of the CD4^+^ T cells in both anatomical sites 37. Consequently, to have a number of T cells sufficient for flow cytometry subsets analysis, the cells isolated from the large and small intestinal biopsies were combined together. In particular, we measured, at baseline, the CD4^+^ and CD8^+^ T‐cell frequencies in peripheral blood and GALT and the frequencies of particular subsets of memory T‐cells. We found no statistical difference in CD4^+^ T‐cells frequencies between peripheral blood and GALT (39.5; IQR 26.9–52.8 and 45.7; IQR 40.9–63.04, respectively; *P* = 0.5). As expected, most of the CD4^+^ T‐cells in GALT showed a central memory (CM: CD4^+^CD27^+^CD45RO^+^) phenotype (85.2, IQR 82.9–86.3) and their percentage was higher than in peripheral blood (47.3, IQR 34.5–61.2; *P* = 0.005); the frequencies of effector memory (EM: CD4^+^CD27^−^CD45RO^+^) CD4^+^ T‐cells were similar in peripheral blood and GALT (7.5; IQR 5.7–12.6 and 10.9; IQR 8.5–13.2, respectively; *P* = 0.2).

CD8^+^ T‐cell frequencies were similar in peripheral blood and GALT (54.8; IQR 40.4–63 and 44.7; IQR 36.9–49.5, respectively; *P* = 0.14); the percentage of CM CD8^+^ T‐cells (CD8^+^CD27^+^CD45RO^+^) was higher in GALT than peripheral blood (78.7; IQR 72.6 and 80; 26.1; IQR 20.7–41.3, respectively; *P* = 0.0001); EM CD8^+^ T (CD8^+^CD27^−^CD45RO^+^) cells frequencies were similar in peripheral blood and GALT (17.4; IQR 10.6–28.7 and 13.11; IQR 10.6–16.1; *P *= 0.2).

### Changes of T‐cell activation in peripheral blood after 6 months of probiotic supplementation

**Figure 2:**
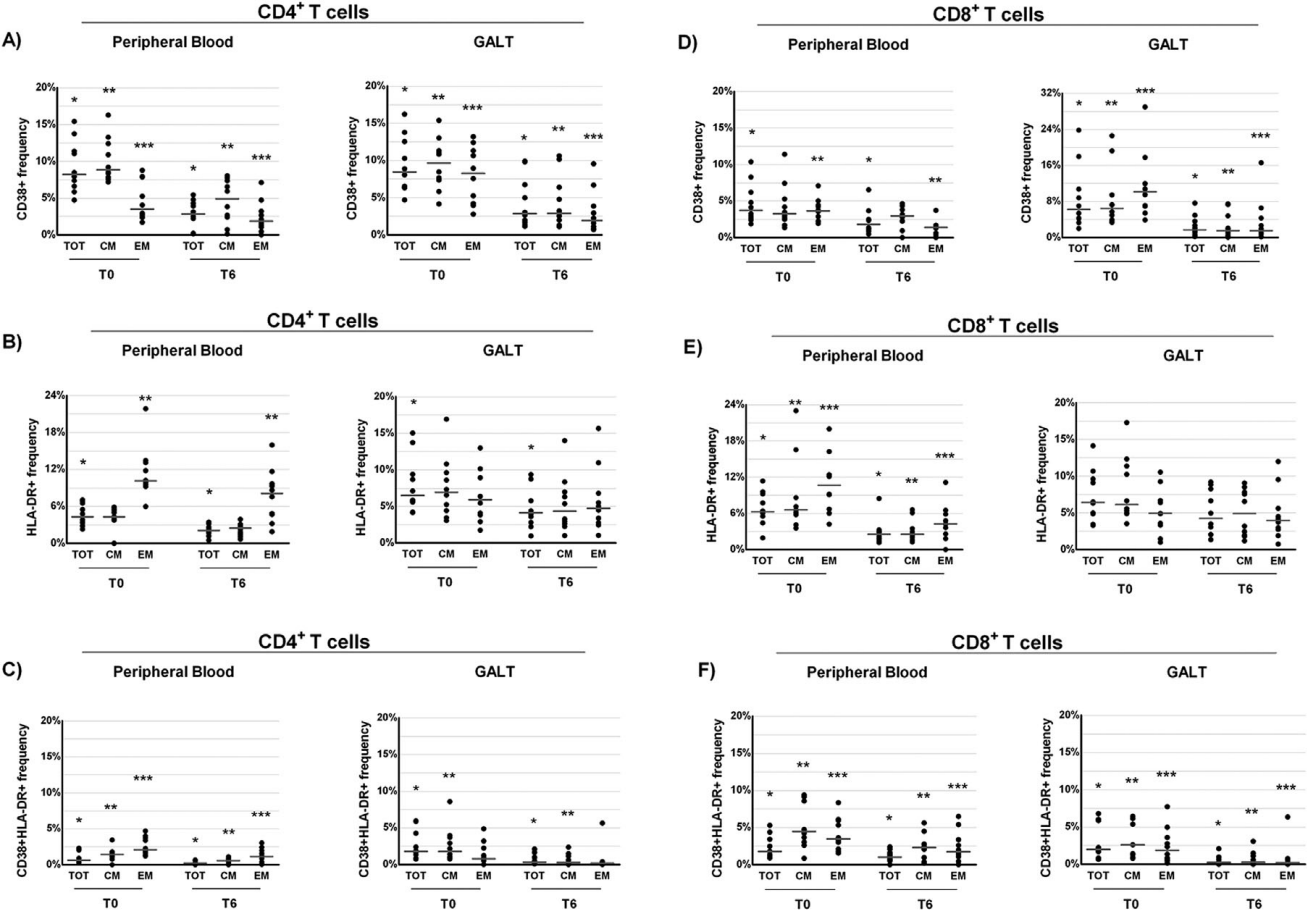
Percentage of CD4^+^ (A–C) and CD8^+^ T (D–F) cell subsets (total [TOT], central memory [CM], and effector memory [EM]) expressing CD38^+^ and HLA‐DR^+^ (CD38^+^ HLA‐DR^−^, CD38^+^ HLADR^+^, and CD38^−^ HLA‐DR^+^) derived from peripheral blood and gut‐associated lymphoid tissue (GALT) of ART‐treated HIV‐1‐positive patients (*n* = 10) before (T0) and after 6 months (T6) of probiotic supplementation. Dot plots represent the frequencies of CD4^+^ T‐cell subsets that express CD38^+^ (A), HLA‐DR^+^ (B), or both (CD38^+^HLA‐DR^+^, Panel C) in peripheral blood and in GALT (Wilcoxon test for paired samples *, **, and *** = *P *< 0.05). Dot plots represent the frequencies of CD8^+^ T‐cell subsets that express CD38^+^ (D), HLA‐DR^+^ (E), or both (CD38^+^HLA‐DR^+^, Panel F) in peripheral blood and in GALT (Wilcoxon test for paired samples ∗, ∗∗ and ∗∗∗ = *P *< 0.05).

The three possible expression patterns of the T‐cell activation markers HLA‐DR^+^ and CD38^+^ (CD38^+^HLA‐DR^−^, CD38^‐^HLA‐DR^+^, and CD38^+^HLA‐DR^+^) were analyzed in CD4+ or CD8^+^ T‐cell subsets from peripheral blood of HIV‐1‐infected patients before and after probiotic supplementation. As seen from Figure 2A, B, D, and E, the frequencies of CD4^+^ and CD8^+^ T‐cells expressing alternately CD38 or HLA‐DR were significantly lower after probiotic supplementation (respectively CD4^+^ T‐cells: *P* = 0.005 and *P* = 0.005; CD8^+^ T‐cells: *P* = 0.037 and *P* = 0.005). Also the proportion of CD4^+^ or CD8^+^ T‐cells simultaneously expressing HLA‐DR and CD38 significantly decreased after 6 months of probiotic supplementation (CD4^+^ T‐cells: *P* = 0.008 and CD8^+^ T‐cell: *P* = 0.037; Fig. 2C and F). Similar results were obtained comparing the frequencies of activated EM CD4^+^ or CD8^+^ T‐cell subsets (Fig. 2A–F). Additionally, a significant reduction of CM CD4^+^CD38^+^HLA‐DR^−^ and CM CD4^+^CD38^+^HLA‐DR^+^ T‐cells at T6 was noticed (*P* = 0.007 and *P* = 0.037, respectively; Fig. 2A and C). Lastly, the CM CD8+ T‐cells with the CD38^‐^HLA‐DR^+^ or CD38^+^HLA‐DR^+^ phenotype also decreased after probiotic supplementation (*P* = 0.007 and *P* = 0.021, respectively) (Fig. 2E and F).

### Changes of T‐cell immune activation in GALT after 6 months of probiotic supplementation

Subsequently, we evaluated the frequencies of CD4+ or CD8^+^ T‐cell subsets that expressed CD38^+^, HLA‐DR^+^, or both to determine whether a reduction of activated T‐cells occurred in GALT after probiotic supplementation. We found a significant reduction in the frequencies of CD4^+^ and CD8^+^ T‐cells expressing alternately CD38^+^ HLA‐DR^+^ or both after probiotic supplementation (*P* = 0.005 for all the comparisons, Fig. 2A–F). The only exception being the frequencies of CD8^+^CD38^‐^HLA‐DR^+^ T‐cells at T6, which although was lower compared to those recorded at T0, the difference did not reach the statistical significance (*P* = 0.139, Fig. 2E). The percentage of EM CD4^+^ T‐cell subpopulations also decreased (*P* = 0.037, Fig. 2A–C) as well as the percentages of EM CD4^+^ T‐cell expressing the CD38^+^ activation marker after probiotic supplementation (*P* = 0.005; Fig. 2A).

Additionally, the frequencies of CM and EM CD8^+^ T‐cells subpopulations with CD38^+^ or CD38^+^HLA‐DR^+^ phenotype were also significantly lower after probiotic assumption (CM: *P* = 0.005 and *P* = 0.005; EM: *P* = 0.005 and *P* = 0.038, respectively; Fig. 2D and F).

### Changes of Th17, Th1, Tc17, and Tc1 frequencies in peripheral blood and GALT after 6 months of probiotic supplementation

**Figure 3:**
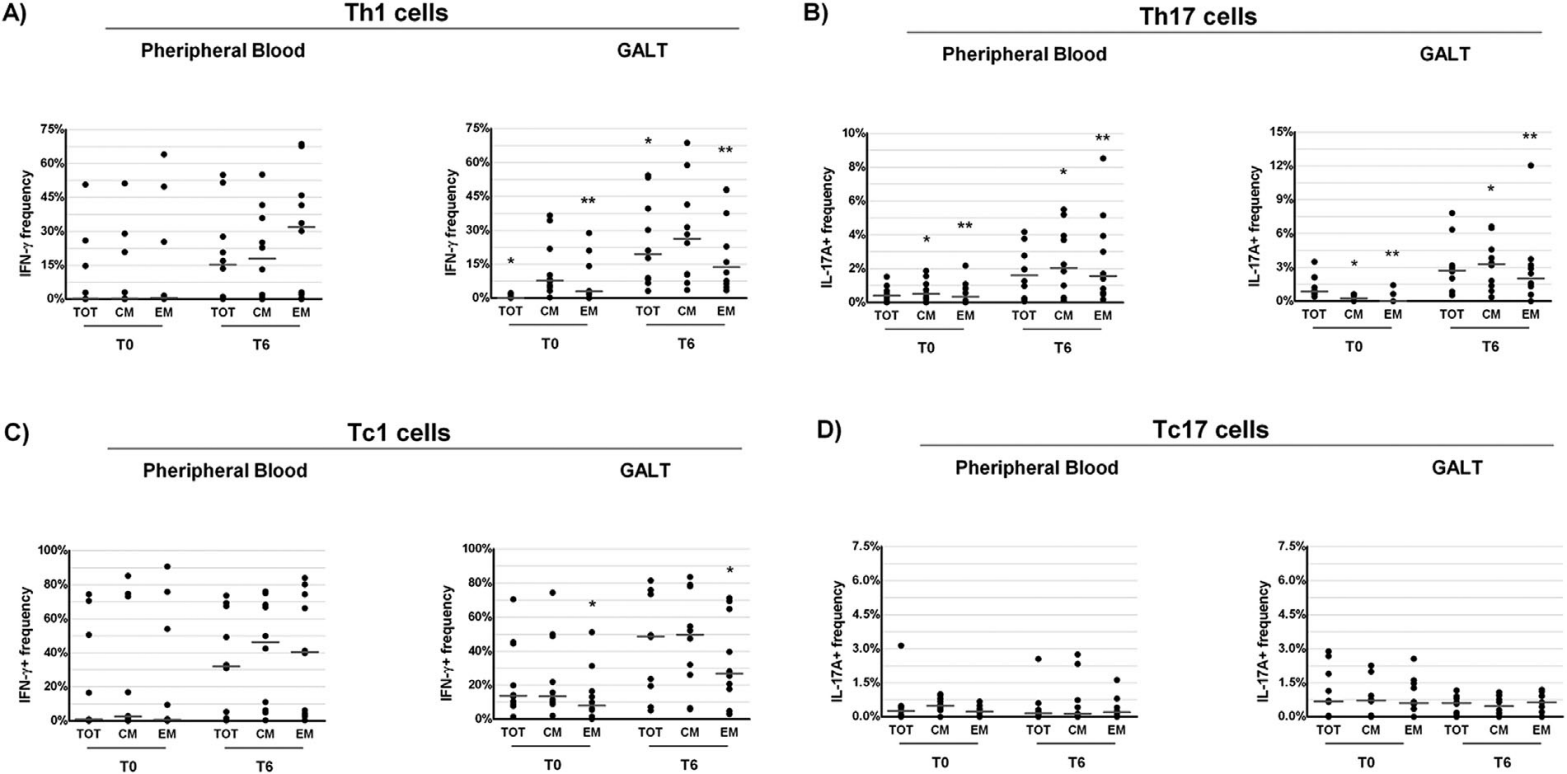
Percentage of CD4^+^ and CD8^+^ T‐cell subsets (total [TOT], central memory [CM], and effector memory [EM]) expressing IFNγ (Th1 and Tc1) and IL‐17A (Th17 and Tc17) derived from peripheral blood and gut‐associated lymphoid tissue (GALT) of ART‐treated HIV‐1‐positive patients (*n* = 10) before (T0) and after 6 months (T6) of probiotic supplementation. Dot plots represent the frequencies of expression of IFNγ (Th1, Panel A) and IL‐17A (Th17, Panel B) CD4^+^ T‐cell subsets in peripheral blood and in GALT (Wilcoxon test for paired samples *, **, and *** = *P* < 0.05). Dot plots represent the frequencies of expression of IFNγ (Tc1, Panel C) and IL‐17A (Tc17, Panel D) CD8^+^ T‐cell subsets in peripheral blood and in GALT (Wilcoxon test for paired samples *, **, and *** = *P* < 0.05).

To assess whether probiotic supplementation promote a recovery of Th17 cells, we measured the frequencies of Th17 in peripheral blood and GALT at T0 and T6. We found a trend toward an increase in the percentage of total Th17 cells both in peripheral blood and GALT after probiotic supplementation (respectively *P* = 0.059 and *P* = 0.059; Fig. 3B). However, a significant improvement in the frequencies of CM and EM Th17 cells was recorded at T6 both in both the anatomical sites (peripheral blood: *P* = 0.028 and *P* = 0.037, respectively; GALT: *P* = 0.007 and *P* = 0.011, respectively, Fig. 3B). Then, we examined the changes in the relative frequencies of Th1, Tc17, and Tc1 cells subsets before and after probiotic supplementation. No significant differences between T0 and T6 were noted in the percentage of Th1, Tc17, and Tc1 cells in peripheral blood (Fig. 3A, C, and D). However, the percentage of EM Tc1 was higher at T6 respectively in the GALT and peripheral blood (*P* = 0.013, *P* = 0.005, respectively; Fig. 3C).

Conversely, a strong increase of total Th1 cells was observed at T6 in GALT (*P* = 0.005, Fig. 3A) and, again, higher relative frequencies of CM and EM, Th1 cell subsets were noted in this anatomical site (*P* = 0.059, *P* = 0.047, and *P* = 0.047, respectively; Fig. 3A).

### Changes in gut histology and morphology after 6 months of probiotic supplementation

**Figure 4:**
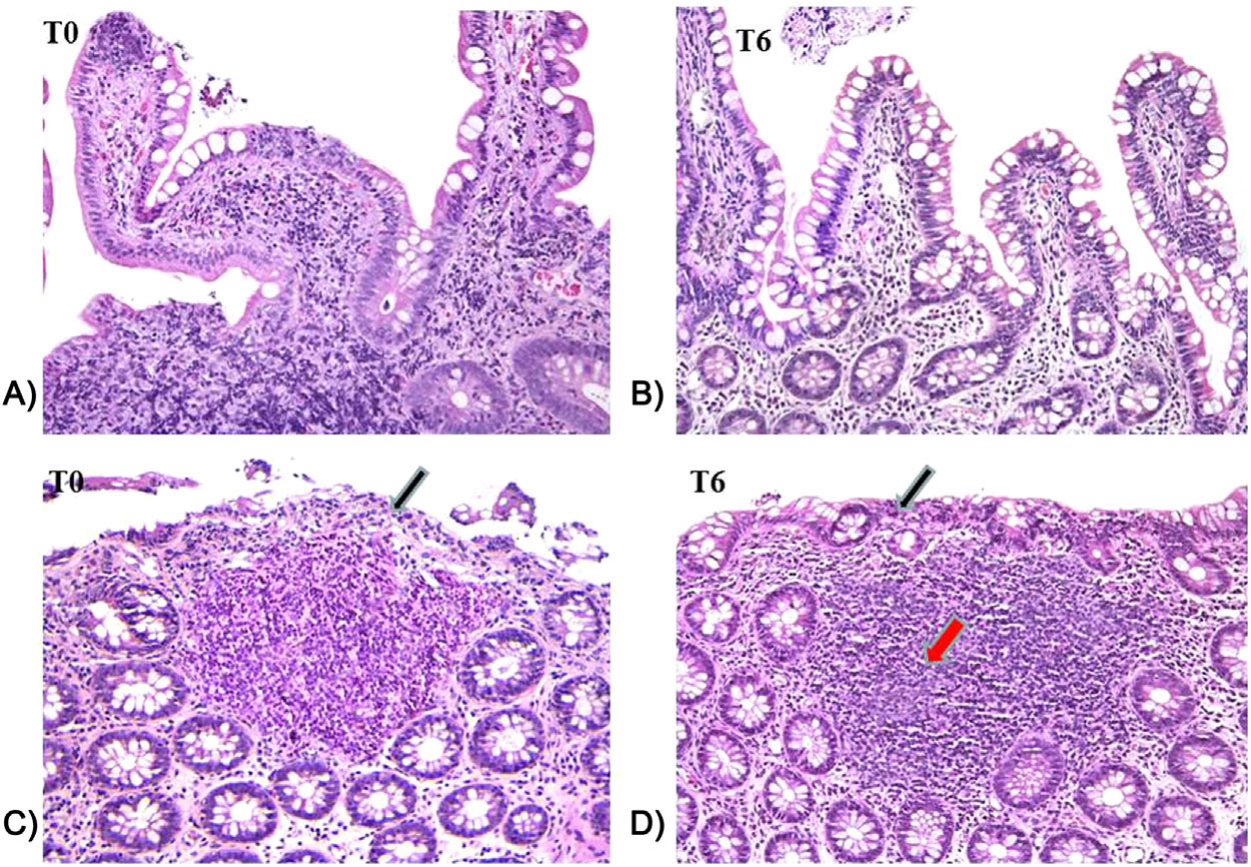
Histological section of descending colon (A and B) and ileum (C and D) from HIV‐1‐positive patients before (T0) and after 6 months of probiotic supplementation (T6). A decrease in the interstitial inflammatory infiltrate and a restoring of the intestinal and colonic epithelium integrity were recorded at T6 (see arrows). 10× enlargement.

**Figure 5:**
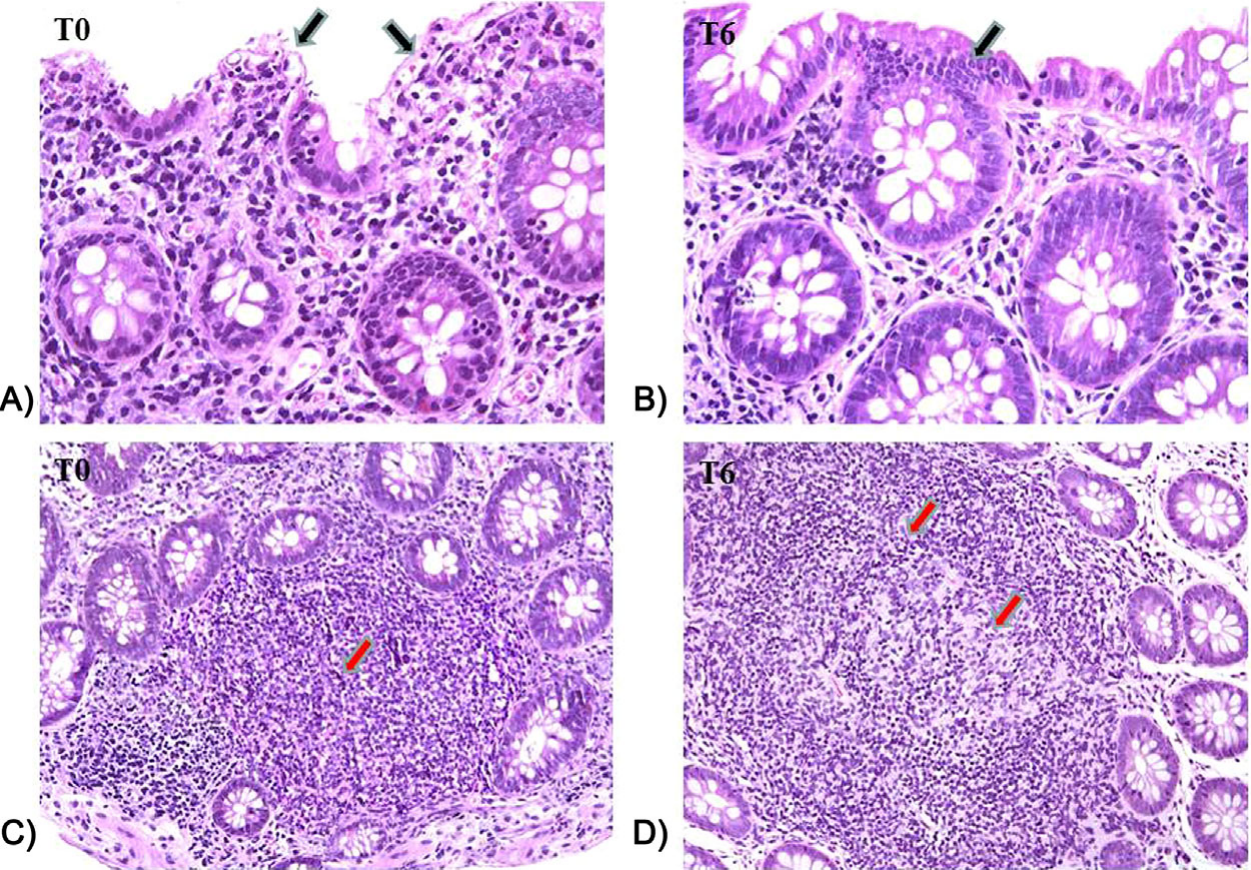
Histological section of descending colon and ileum from HIV‐1‐positive patients before (T0) and after 6 months of probiotic supplementation (T6). An improvement of colonic epithelium integrity (see black arrows) and a decrease in the lymphocyte transcytosis (A and B) were recorded at T6. A strong expansion of the Peyer patches (see red arrow; C and D) and the appearance of the typical (clear area) was recorded in the ileum at T6. 20× enlargement.

**Figure 6:**
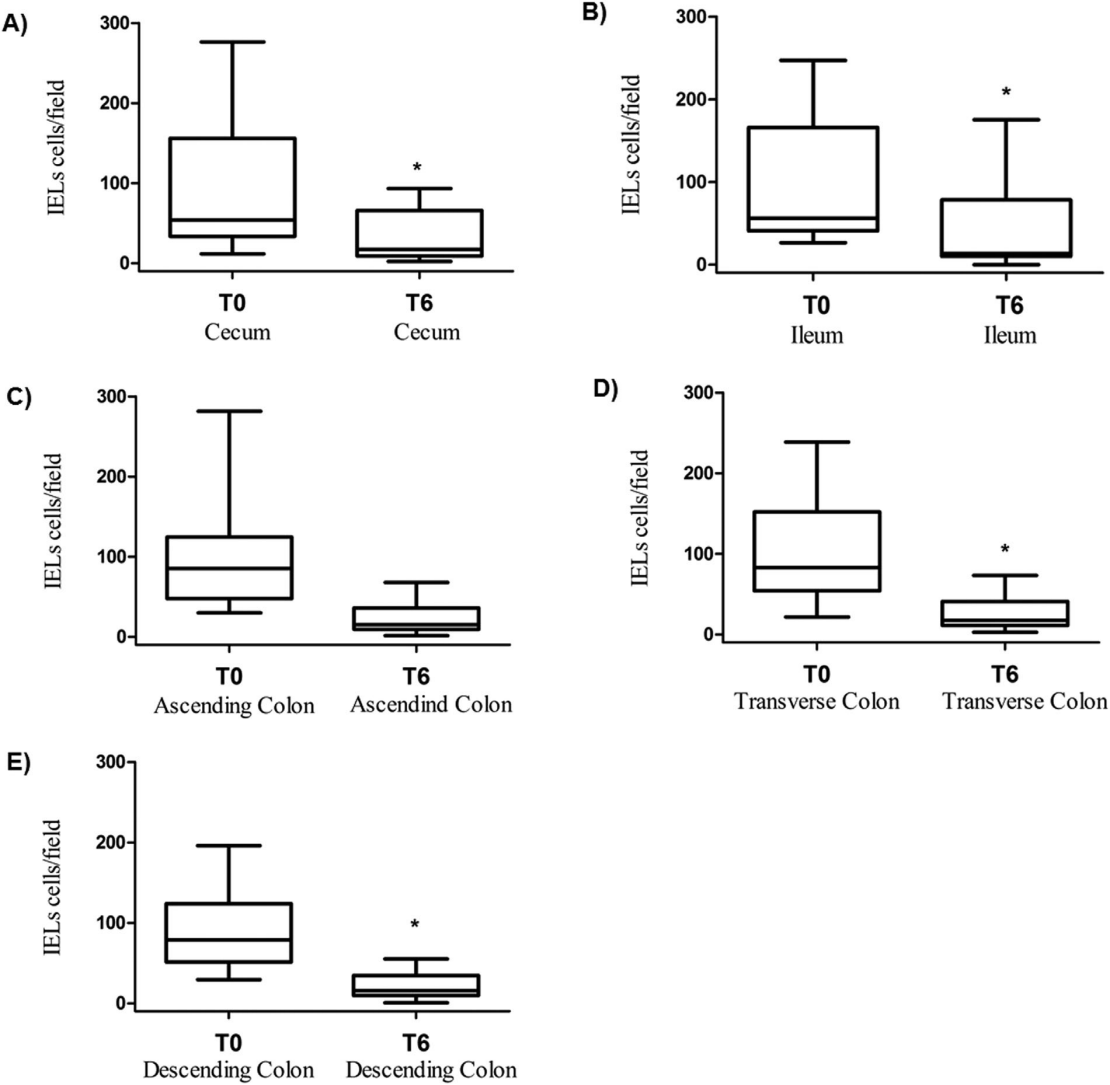
Intraepithelial lymphocytes (IELs) density in cecum (A), ileum (B), ascending (C), transverse (D), and descending colon (E) biopsies from HIV‐1‐infected patients (*n* = 10) who achieved virological suppression in response to ART therapy at enrollment (T0) and after 6 months (T6) of probiotic supplementation. The results shows a significant reduction of IELs density in cecum, ascending, transverse, and descending colon (*T0 vs. T6; *P *< 0.05).

The observation of increased frequencies of intestinal Th17 cells and decreased T‐cell activation after supplementation raises the possibility that probiotic administration might also promote the reversing of gut damage in HIV‐1 infection. To directly pursue this possibility, we performed a histologic and morphologic analysis of GALT and intestinal tract tissues before and after probiotic supplementation. We found that the histology score, evaluated before the beginning of probiotic assumption (T0) and after 6 month (T6), generally decreased as demonstrated in Figures 4 and 5. Remarkably, from a morphological point of view, after the probiotic administration, sections of all tracts of intestinal mucosa, regardless of the examined intestine tract, showed an improvement of epithelial integrity, a reduction of diffuse interstitial inflammatory infiltrate, and an increase in the number and area of the GALT structures associated to the mucosa (Figs. 4 and 5). Then, we evaluated the inflammatory infiltrate in intestinal biopsies collected before and after the probiotic supplementation, focusing on intraepithelial lymphocytes (IELs) density. A general decline in IELs counts in all intestinal tracts analyzed after probiotic supplementation was recorded (Fig. 6). In particular, the number of IELs was significantly lower at T6 compared to those at T0 in the cecum (Fig. 6A), ileum (Fig. 6B), transverse (Fig. 6D), descending colon (Fig. 6E) (*P* = 0.049, *P* = 0.027, *P* = 0.004, and *P* = 0.002, respectively). A trend toward a reduction in IELs numbers after probiotic supplementation was also observed in the ascending colon but the difference did not reach statistical significance (*P* = 0.06, Fig. 6C).

### Changes of enterocytes apoptosis and HSP60 levels after 6 months of probiotic supplementation

**Figure 7:**
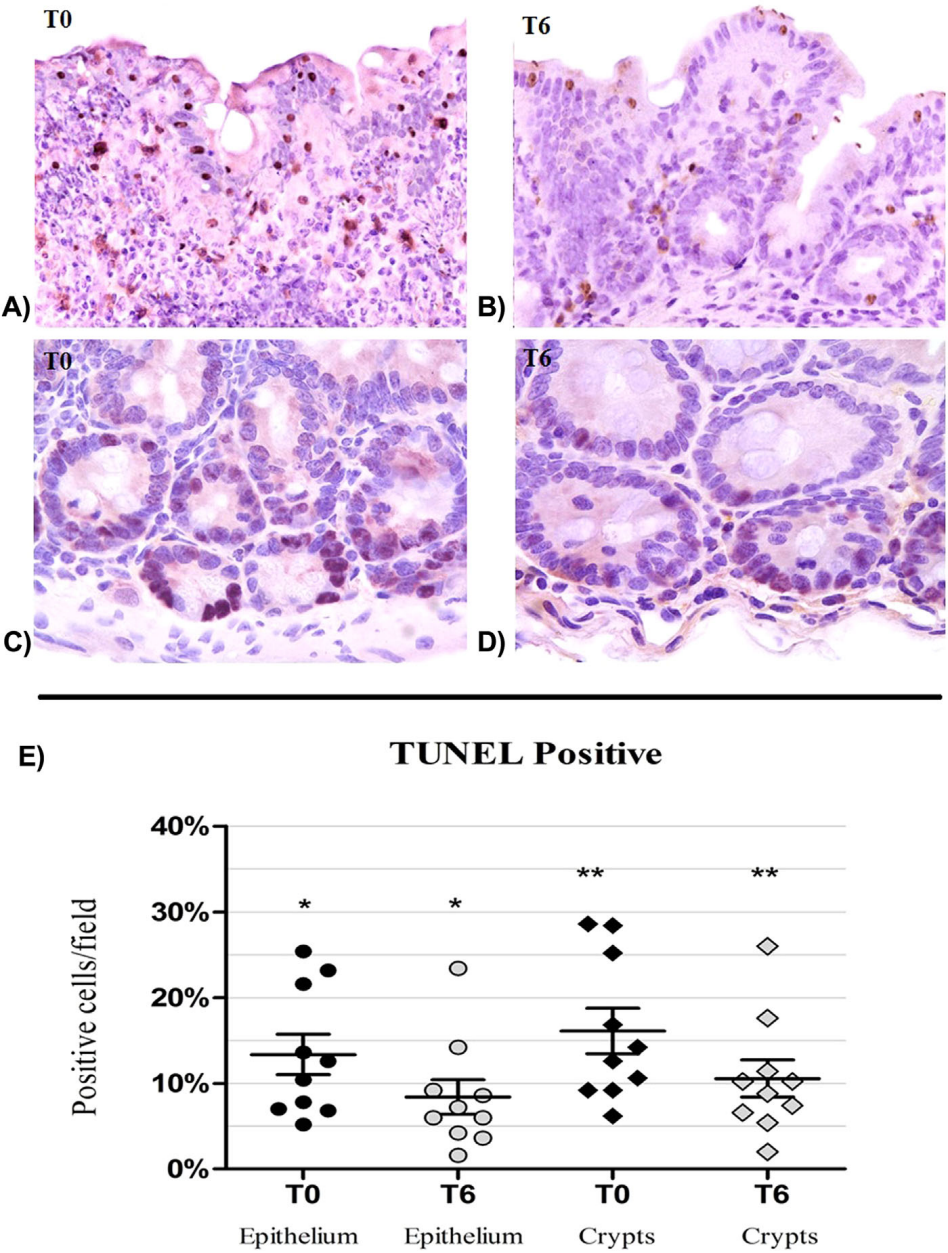
TUNEL apoptosis in HIV‐1‐infected patients before (T0) and after 6 (T6) months of probiotic supplementation. (A and B) Apoptosis in the colon epithelium and in lymphocytes infiltrating epithelia at T0 and T6 (10× enlargement). (C and D) Apoptotic nuclei in colonic crypts cells at T0 and T6 (20× enlargement). (E) Detection of apoptosis in mucosal epithelial surface and in the crypts. A significant reduction of apoptosis is observed in both epithelium and crypts at T6 (*T0 vs. T6, *P* < 0.05, Wilcoxon test).

**Figure 8:**
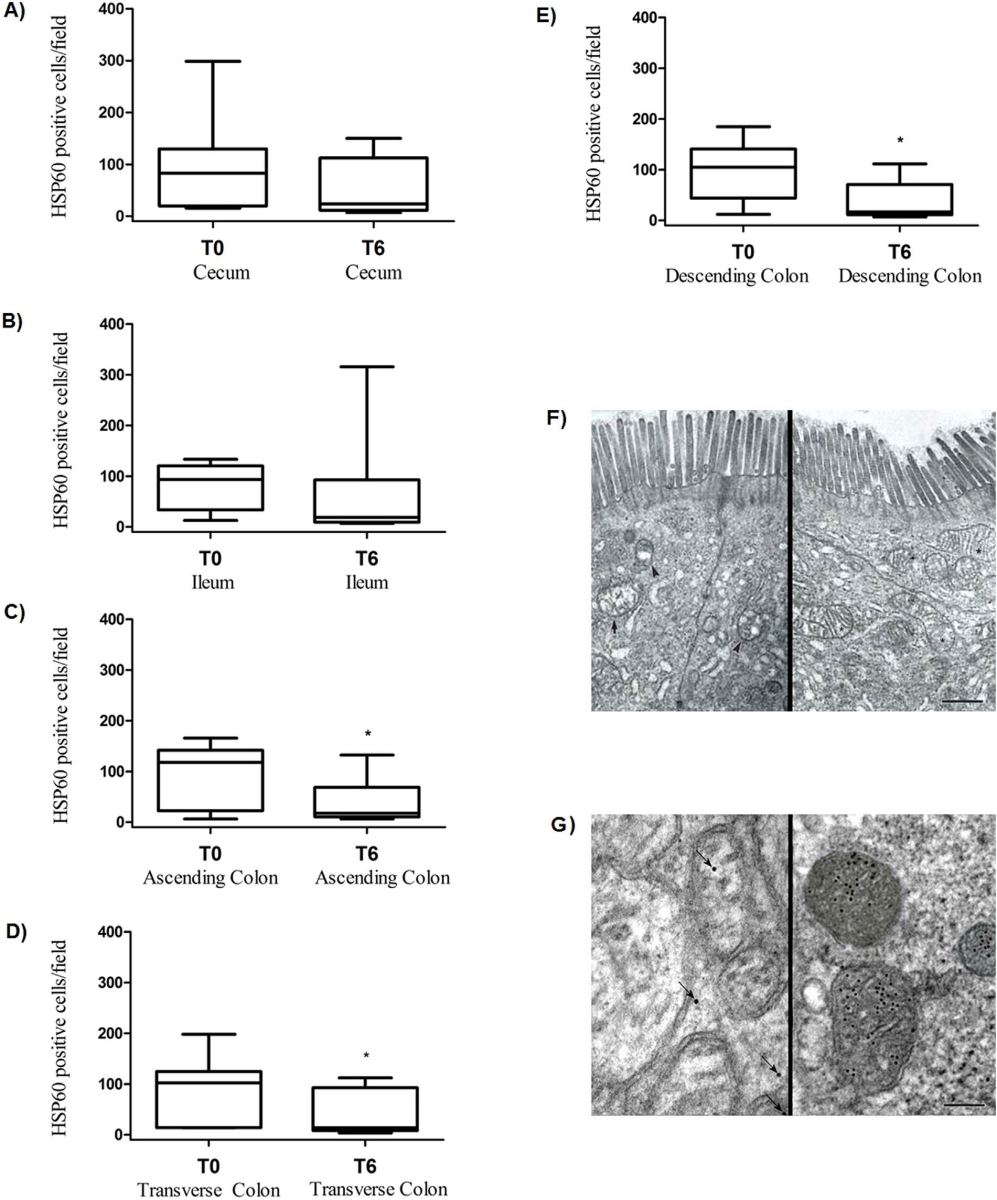
Gut HSP60 expression and TEM evaluation of ileum epithelium before (T0) and after 6 months (T6) of probiotic supplementation. HSP60‐positive cells/field in cecum (A), ileum (B), ascending (C), transverse (D), and descending (E) colon biopsies at T0 and T6 *P < 0.05 (T0 vs. T6) in ascending, descending, and transverse colon biopsies. (F) The mitochondrial component is highly edematous, bulgy, with cristolysis areas (see arrow) and vacuolations (see arrows head) at T0. A recovery of the mitochondrial structures (see asterisk) was recorded at T6 (scale bar = 500 mm). (G) Immunogold in the ileum sections: anti‐HSP60 antibody‐conjugated gold particles are scarcely present in mitochondrial and they are located in extra‐mitochondrial space at T0 (see arrows), conversely anti‐HSP60 antibody conjugated gold particles are located in large amount inside the mitochondria at T6 (scale bar = 100 nm).

Given that this specific probiotic supplementation seemed to induce a recovery of intestinal integrity of epithelial barrier and a reduction in IELs density, we examined whether probiotic supplementation leads to reduce rate of enterocytes undergoing apoptosis. As shown in Figure 7, we found that the decline of IELs infiltrating the intestinal epithelium after the probiotic supplementation was strictly associated to a statistically significant decrease in the levels of apoptosis index both in epithelium and intestinal crypts (*P* = 0.04). Additionally, a decrease in the enterocyte levels of cytoplasmic HSP60 protein was noticed. Indeed, comparing the HSP60 levels in intestinal mucosa before and after the probiotic assumption, a trend toward a reduction of HSP60 was found in cecum (Fig. 8A, T0: 82.70/19.85–129.8, T6: 23.80/11.65–112.20, *P* = 0.432) and ileum (Fig. 8B, T0: 93.40/33.65–120.4, T6: 18.70/9.45–92.80, *P* = 0.275), while the HSP60 median values decreased significantly after probiotic supplementation in ascending (Fig. 8C), transverse (Fig. 8D), and descending colon (Fig. 8E) (*P* = 0.01; *P* = 0.037; *P* = 0.04, respectively).

At the same time, all the intestinal sections evaluated by TEM, demonstrated that enterocytes regeneration after probiotic supplementation was associated to a good preservation of mitochondrial structure, and that the reduction in intracytoplasmic expression of HSP60 was related to a strong increase of intra‐mitochondrial concentration of this protein (Fig. 8F and G). In particular, HSP60 was detected closely related to the inner membrane, or free in mitochondrial matrix, adjacent to the mitochondrial cristae; at the same time, HSP60 was not detectable in a free intracytoplasmic pattern inside enterocytes belonging to different portions of intestinal mucosa. Furthermore, the examined mitochondria were almost always found in the “orthodox” conformation, characterized by a relatively large matrix volume and the inner boundary membrane (the non‐cristae component of the inner membrane) closely opposed to the outer membrane with a small space between them.

## Discussion

Given the contributions of microbial dysbiosis and preferential depletion of Th17 cells to GI tract injury, microbial translocation, and persistent immune activation 38, 39, 40, we considered that supplementing ART therapy in HIV‐1‐infected patients with probiotic mixture might favor a restoration of the composition of the commensal gut microbiota, beginning a sequence of immune and gut physiology restorative processes. We assessed longitudinally the effects of the probiotic supplementation on several aspects of gut mucosal immunity in ART‐treated HIV‐1‐patients with viral suppression before and after 6 months of probiotic supplementation. Moreover, in order to ascertain the effectiveness of this probiotics in reversing intestinal barrier injury, we performed a detailed histological and morphological evaluation of GALT and intestinal tract tissues. We found that: (i) a significant reduction of T‐cell activation occurs both in peripheral blood and in the GALT; (ii) the frequency of CM and EM Th17 cell subsets increases especially in GALT but also in the peripheral blood while Tc17 frequencies remained unchanged; (iii) the probiotic improved the integrity of the gut epithelial barrier; (iv) the probiotic supplementation leads to a reduction of both IELs density and enterocytes death via apoptosis; and (v) the probiotic improved mitochondrial morphology trough a localization of mitochondrial chaperonin, HPS60, from cytosol to mitochondrial compartment. Last but not least, consistent with our previous study, we found a clear and significant reduction in the levels of systemic immune activation on CD4^+^ or CD8^+^ T‐cell subsets, defined by CD38^+^ and HLA‐DR^+^ markers, after probiotic supplementation 15.

These findings reinforced the concept on the beneficial impact of this specific probiotic intervention on systemic immune activation in chronically HIV‐1‐infected patients. In this pilot study, we also demonstrated that the reduction of T‐cell activation occurs also in the GALT compartment, indicating that the probiotic intervention is not associated to an increase of intestinal mucosal inflammation but to a reduction of the harmful excessive activation of T‐cell immunity in this anatomical site, in agreement with what recently demonstrated in the colon of in healthy macaques post probiotic therapy 41. Remarkably, the reduction of T‐cell activation was accompanied to a strong increase in the percentage of CM and EM Th17 cells after probiotic supplementation in both the anatomical sites analyzed. This increase was particularly evident in the GALT for the CM Th17 subset (more than 10‐fold increase) compared to peripheral blood (fivefold increase). Moreover, we also found that the probiotic intervention increased the percentage of Th1 cell subsets in GALT. The discrepancy observed between GALT and PBMC compartments in terms of CD4^+^ T‐cell subsets increased after the probiotic intake reflects the complexity of gut T‐cell depletion observed during HIV‐1 infection. It is known that direct and indirect viral cytopathic effects, host‐derived cellular immune response, activation‐induced cell death and, perhaps, alterations in mucosal homing, may occur simultaneously. Also previous studies have shown that circulating T‐cell subset and tissue‐specific lymphocyte migration patterns can occur independently 42, 43, 44, 45. These increased reconstitutions after probiotic therapy are important, considering that long‐term ART‐treated HIV‐1‐infected patients seldom reconstitute GI tract CD4^+^ T‐cells to healthy levels 46 probably a cause of alterations in CD4^+^ T‐cell homing to the gut 47. In agreement, Klatt et al. 11 have shown that the probiotic supplementation of SIV‐infected pigtail macaques leads to gut CD4^+^ T‐cell restoration and enhanced Th17 functionality. However, the improvement in the frequency of Th17 cells seems to be different in SIV infection models depending on whether the probiotic was supplemented with IL‐21 or not 11, 12. Conversely to that observed on the Th17 levels, we found the probiotic intervention did not affect the percentage of Tc17 in GALT and peripheral blood. These cells are maintained in the gut of RMs during acute SIV infection, but they are progressively depleted during end‐stage disease 48, 49. In order to have a more detailed view about the action of this probiotic formulation on gut mucosa, a histomorphological analysis was performed in multiple intestinal tract tissues biopsies taken from the HIV‐1‐positive patients before and after probiotic supplementation. It is known that histopathologic changes during HIV‐1‐related enteropathy include villous blunting and widening, vacuolated enterocytes, crypt hyperplasia, and increased inflammatory cells in the lamina propria 50, 51, 52, 53. Similar histopathologic alterations were observed in all the HIV‐1‐positive patients before probiotic intervention. The probiotic supplementation caused histomorphological and ultrastructural changes of all intestinal tract tissues analyzed leading to an improvement of epithelial integrity, a reduction of diffuse interstitial inflammatory infiltrate, and an increase in the number and area of the GALT structures. Moreover, a strong reduction in IELs density was also observed. Notably, Mattapallil et al. [54] reported that that IELs can support the active replication of SIV and that their phenotypic profile and function were changed in SIV infection. The latter aspects could have several implications on the HIV‐1‐associated enteropathy and indirectly on the beneficial effects of probiotic observed on the intestinal immunity and epithelium barrier repair. Indeed, since the functions carried out by IELs in the gut mucosa seem to be mediated in part by the cytokines (i.e., IFNγ, tumor necrosis factor alpha [TNFα], and interleukin‐2 [IL‐2]) and chemokines (MIP‐1β and RANTES) that they produce, these cells may play a relevant role in regulating the intestinal cytokine network of HIV‐1‐infected patients contributing, in a situation of functional alteration, to the breakdown of the intestinal immunity 54, 55. As a matter of fact, high levels of TNFα are thought to induce enterocytes apoptosis and supplementation with anti‐TNF antibodies is of important clinical benefit to patients suffering from Crohn's disease 56. Directly related to this aspect, another important finding of our study was that enterocytes apoptotic index was found to be considerably downregulated after probiotic supplementation, indicating a stimulating probiotic action on gut regrowth and re‐epithelization. In agreement, Mogilner et al. 57 found that probiotic supplementation causes a decrease in enterocyte apoptosis in a rat model with massive small bowel resection. The mechanisms underlying the effects of probiotic on the enterocytes apoptosis are currently unknown. However, the enterocytes apoptosis rate in HIV‐1‐positive patients has been related to the HIV‐1 transactivator factor (Tat), through an oxidative stress mediated mechanism 58 and an antioxidant effects of probiotic for contrasting the adverse effects of reactive oxygen species (ROS) on intestinal barrier has been reported 59. Alongside the effects of probiotic on reducing IELs density and enterocytes apoptosis, we found that epithelial barrier amelioration was strictly related to a mitochondrial morphological improvement after probiotic supplementation, and a strong HSP60 expression was evidenced into mitochondrial matrix by immunogold technique, associated to a decrease, in absolute sense, in intracytoplasmic concentration. Interestingly, the cytosolic accumulation of HSP60 has been shown to represent an ordinary event during apoptosis induction and that the pro‐death role of HSP60 seems to require caspase‐3 maturation and cytoplasmic activation 60. Moreover, the presence of HSP60 in mitochondria of the gastrointestinal tract has been associated to the following mechanisms of organelle renewal: (i) total reformation of mitochondria and their content after cell division and (ii) regeneration of mitochondrial after ATP production 61. There were any studies on the evaluation of HSP60 in intestinal tracts of HIV‐1‐infected patients; however, in agreement to our finding, it has been showed that HSP60—classically a mitochondrial protein—was abundantly also present in cytosol in intestinal biopsies taken at the time of diagnosis from patients suffering from ulcerative colitis, but not after the probiotic supplementation 62.

Although limited in the sample size of HIV‐1‐infected patients analyzed due to the necessity to evaluate the implementation of the novel intervention with a high concentration multi‐strain probiotic in terms of safety, tolerance, and efficacy, our study represent an important incentive to justify larger randomized double bind clinical studies in order to characterize in detail the mechanism of the probiotic action on both mucosal and systemic immunity, GI physiology, and viral reservoir and to assess whether probiotic intervention could improve the prognosis of ART‐treated HIV‐1‐infected individuals. It will also be important to evaluate whether the improved immunophenotypic pattern observed after probiotics treatment remained stable after cessation of probiotic intake.

At the same time, it should be emphasized that probiotics are normally made up of a genus (e.g., *Streptococcus*), a species (e.g., *thermophilus*), and a strain (e.g., DSM24731); while the genus and species tells us a bit about its properties, it is the probiotic strain itself which determines the efficacy of the product and hence the associated findings. Accordingly, it should be reminded that the results of our pilot study should not be translated or generalized to other probiotic formulations differing for number of live/dead bacteria, strains type or manufacturing processes, especially when the target population is at risk like ART subjects.

## Conflict of Interest

None declared.

## Supporting information

Additional supporting information may be found in the online version of this article at the publisher's web‐site.

**Figure S1**. Consort checklist.

**Figure S2**. Flow diagram.

**Figure S3**. Gating strategy for flow cytometry analysis of peripheral blood and GALT. Singlets were identified by FSC‐A versus FSC‐H dot plot (A). After gating on lymphocytes, identified by the FSC and SSC parameters (B), CD3+CD4+ and CD3+CD8+ gates were defined (C and D); for both of them, central memory (CM) and effector memory (EM) gates were identified by the expression of CD45ro and CD27 (E). CD8 and CD4 lymphocytes and the CM and EM subpopulations were investigated for the activation status by the CD38 and HLA‐DR expression (F) and for the intra‐citoplasmatic expression of IFN‐γ (Th1 and Tc1, respectively) and IL‐17A (Th17 and Tc17, respectively) (G).

## References

[1] Marchetti, G. , C. Tincati , and G. Silvestri . 2013 Microbial translocation in the pathogenesis of HIV infection and AIDS. Clin. Microbiol. Rev. 26:2–18. 23297256 10.1128/CMR.00050-12 PMC3553668

[2] Sandler, N. G. , and D. C. Douek . 2012 Microbial translocation in HIV infection: causes, consequences and treatment opportunities. Nat. Rev. Microbiol. 10:655–666. 22886237 10.1038/nrmicro2848

[3] Klatt, N. R. , and J. M. Brenchley . 2010 Th17 cell dynamics in HIV infection. Curr. Opin. HIV AIDS 5:135–140. 20543590 10.1097/COH.0b013e3283364846 PMC2886291

[4] Estes, J. D. , L. D. Harris , N. R. Klatt , B. Tabb , S. Pittaluga , M. Paiardini , G. R. Barclay , J. Smedley , R. Pung , and K. M. Oliveira , et al. 2010 Damaged intestinal epithelial integrity linked to microbial translocation in pathogenic simian immunodeficiency virus infections. PLoS Pathog. 6:e1001052. 20808901 10.1371/journal.ppat.1001052 PMC2924359

[5] Paiardini, M. 2010 Th17 cells in natural SIV hosts. Curr. Opin. HIV AIDS 5:166–172. 20543595 10.1097/COH.0b013e328335c161

[6] Cecchinato, V. , and G. Franchini . 2010 Th17 cells in pathogenic simian immunodeficiency virus infection of macaques. Curr. Opin. HIV AIDS 5:141–145. 20543591 10.1097/COH.0b013e32833653ec PMC2898129

[7] Dillon, S. M. , E. J. Lee , A. M. Donovan , K. Guo , M. S. Harper , D. N. Frank , M. D. McCarter , M. L. Santiago , and C. C. Wilson . 2016 Enhancement of HIV‐1 infection and intestinal CD4+ T cell depletion ex vivo by gut microbes altered during chronic HIV‐1 infection. Retrovirology 13:5. 26762145 10.1186/s12977-016-0237-1 PMC4712466

[8] Lozupone, C. A. , M. Li , T. B. Campbell , S. C. Flores , D. Linderman , M. J. Gebert , R. Knight , A. P. Fontenot , and B. E. Palmer . 2013 Alterations in the gut microbiota associated with HIV‐1 infection. Cell Host. Microbe 14:329–339. 24034618 10.1016/j.chom.2013.08.006 PMC3864811

[9] Dillon, S. M. , E. J. Lee , C. V. Kotter , G. L. Austin , Z. Dong , D. K. Hecht , S. Gianella , B. Siewe , D. M. Smith , A. L. Landay , et al. 2014 An altered intestinal mucosal microbiome in HIV‐1 infection is associated with mucosal and systemic immune activation and endotoxemia. Mucosal. Immunol. 7:983–994. 24399150 10.1038/mi.2013.116 PMC4062575

[10] Zevin, A. S. , L. McKinnon , A. Burgener , and N. R. Klatt . 2016 Microbial translocation and microbiome dysbiosis in HIV‐associated immune activation. Curr. Opin. HIV AIDS 11:182–190. 26679414 10.1097/COH.0000000000000234 PMC4752849

[11] Klatt, N. R. , L. A. Canary , X. Sun , C. L. Vinton , N. T. Funderburg , D. N. R. Morcock , M. Quiñones , C. B. Deming , M. Perkins , D. J. Hazuda , et al. 2013 Probiotic/prebiotic supplementation of antiretrovirals improves gastrointestinal immunity in SIV‐infected macaques. J. Clin. Invest. 123:903–907. 23321668 10.1172/JCI66227 PMC3561826

[12] Ortiz, A. M. , Z. A. Klase , S. R. DiNapoli , I. Vujkovic‐Cvijin , K. Carmack , M. R. Perkins , N. Calantone , C. L. Vinton , N. E. Riddick , J. Gallagher , et al. 2016 IL‐21 and probiotic therapy improve Th17 frequencies, microbial translocation, and microbiome in ARV‐treated, SIV‐infected macaques. Mucosal Immunol. 9:458–467. 26286233 10.1038/mi.2015.75 PMC4760912

[13] Vujkovic‐Cvijin, I. , L. A. Swainson , S. N. Chu , A. M. Ortiz , C. A. Santee , A. Petriello , R. M. Dunham , D. W. Fadrosh , D. L. Lin , A. A. Faruqi , et al. 2015 Gut‐resident *Lactobacillus* abundance associates with IDO1 inhibition and Th17 dynamics in SIV‐infected macaques. Cell. Rep. 13:1589–1597. 26586432 10.1016/j.celrep.2015.10.026 PMC4782968

[14] Falasca, K. , J. Vecchiet , C. Ucciferri , M. Di Nicola , C. D'Angelo , and M. Reale . 2015 Effect of probiotic supplement on cytokine levels in HIV‐infected individuals. A preliminary study. Nutrients 7:8335–8347. 26426044 10.3390/nu7105396 PMC4632416

[15] d'Ettorre, G. , G. Ceccarelli , N. Giustini , S. Serafino , N. Calantone , G. De Girolamo , L. Bianchi , V. Bellelli , T. Ascoli‐Bartoli , S. Marcellini , et al. 2015 Probiotics reduce inflammation in antiretroviral treated, HIV‐infected individuals: results of the “probio‐HIV” clinical trial. PLoS ONE 10:e0137200. 26376436 10.1371/journal.pone.0137200 PMC4573418

[16] Stiksrud, B. , P. Nowak , F. C. Nwosu , D. Kvale , A. Thalme , A. Sonnerborg , P. M. Ueland , K. Holm , S. E. Birkeland , A. E. Dahm , et al. 2015 Reduced levels of D‐dimer and changes in gut microbiota composition after probiotic intervention in HIV‐infected individuals on stable ART. J. Acquir. Immune Defic. Syndr. 70:329–3337. 26258571 10.1097/QAI.0000000000000784

[17] Villar‐García, J. , J. J. Hernández , R. Güerri‐Fernández , A. González , E. Lerma , A. Guelar , D. Saenz , L. Sorlí , M. Montero , P. J. Horcajada , et al. 2015 Effect of probiotics (*Saccharomyces boulardii*) on microbial translocation and inflammation in HIV‐treated patients: a double‐blind, randomized, placebo‐controlled trial. J. Acquir. Immune Defic. Syndr. 68:256–263. 25469528 10.1097/QAI.0000000000000468

[18] Yang, O. O. , T. Kelesidis , R. Cordova , and H. Khanlou . 2014 Immunomodulation of antiretroviral drug‐suppressed chronic HIV‐1 infection in an oral probiotic double‐blind placebo‐controlled trial. AIDS Res. Hum. Retroviruses 30:988–995. 25127924 10.1089/aid.2014.0181 PMC6461151

[19] Schunter, M. , H. Chu , T. L. Hayes , D. McConnell , S. S. Crawford , P. A. Luciw , S. Bengmark , D. M. Asmuth , J. Brown , C. L. Bevins , et al. 2012 Randomized pilot trial of a synbiotic dietary supplement in chronic HIV‐1 infection. BMC Complement. Altern. Med. 12:84. 22747752 10.1186/1472-6882-12-84 PMC3414771

[20] Cunningham‐Rundles, S. , S. Ahrné , R. Johann‐Liang , R. Abuav , A.‐M. Dunn‐Navarra , C. Grassey , S. Bengmark , and J. S. Cervia . 2011 Effect of probiotic bacteria on microbial host defense, growth, and immune function in human immunodeficiency virus type‐1 infection. Nutrients 3:1042–1070. 22292110 10.3390/nu3121042 PMC3260491

[21] d'Ettorre, G. , S. Baroncelli , L. Micci , G. Ceccarelli , M. Andreotti , P. Sharma , G. Fanello , F. Fiocca , E. N. Cavallari , N. Giustini , et al. 2014 Reconstitution of intestinal CD4 and Th17 T cells in antiretroviral therapy suppressed HIV‐infected subjects: implication for residual immune activation from the results of a clinical trial. PLoS ONE 9:e109791. 25340778 10.1371/journal.pone.0109791 PMC4207675

[22] Nowak, P. , M. Troseid , E. Avershina , B. Barqasho , U. Neogi , K. Holm , J. R. Hov , K. Noyan , J. Vesterbacka , J. Svärd , et al. 2015 Gut microbiota diversity predicts immune status in HIV‐1 infection. AIDS Lond. Engl. 29:2409–2418. 10.1097/QAD.0000000000000869 26355675

[23] Dinh, D. M. , G. E. Volpe , C. Duffalo , S. Bhalchandra , A. K. Tai , A. V. Kane , C. A. Wanke , and H. D. Ward . 2015 Intestinal microbiota, microbial translocation, and systemic inflammation in chronic HIV infection. J. Infect. Dis. 211:19–27. 25057045 10.1093/infdis/jiu409 PMC4326316

[24] Sanders, M. E. , L. M. A. Akkermans , D. Haller , C. Hammerman , J. Heimbach , G. Hörmannsperger , G. Huys , D. D. Levy , F. Lutgendorff , D. Mack , et al. 2010 Safety assessment of probiotics for human use. Gut Microbes 1:164–185. 21327023 10.4161/gmic.1.3.12127 PMC3023597

[25] Ganji‐Arjenaki M. , and M. Rafieian‐Kopaei . 2017 Probiotics are a good choice in remission of inflammatory bowel diseases: a meta analysis and systematic review. J. Cell Physiol. https://doi.org/10.1002/jcp.25911 [Epub ahead of print]. 10.1002/jcp.25911 28294322

[26] Gionchetti, P. , F. Rizzello , A. Venturi , and M. Campieri . 2000 Probiotics in infective diarrhoea and inflammatory bowel diseases. J. Gastroenterol. Hepatol. 15:489–493. 10847433 10.1046/j.1440-1746.2000.02162.x

[27] Scagnolari, C. , G. Corano Scheri , C. Selvaggi , I. Schietroma , S. Najafi Fard , A. Mastrangelo , N. Giustini , S. Serafino , C. Pinacchio , P. Pavone , et al. 2016 Probiotics differently affect gut‐associated lymphoid tissue indolamine‐2, 3‐dioxygenase mRNA and cerebrospinal fluid neopterin levels inantiretroviral‐treated HIV‐1 infected patients: a pilot study. Int. J. Mol. Sci. 17. 10.3390/ijms17101639 PMC5085672 27689995

[28] Mastromarino, P. , D. Capobianco , G. Campagna , N. Laforgia , P. Drimaco , A. Dileone , and M. E. Baldassarre . 2014 Correlation between lactoferrin and beneficial microbiota in breast milk and infant's feces. Biometals 27:1077–1086. 24970346 10.1007/s10534-014-9762-3

[29] Matsuki, T. , K. Watanabe , J. Fujimoto , T. Takada , and R. Tanaka . 2004 Use of 16S rRNA gene‐targeted group‐specific primers for real‐time PCR analysis of predominant bacteria in human feces. Appl. Environ. Microbiol. 70:7220–7228. 15574920 10.1128/AEM.70.12.7220-7228.2004 PMC535136

[30] IBD Working Group of the European Society for Paediatric Gastroenterology, Hepatology and Nutrition . 2005 Inflammatory bowel disease in children and adolescents: recommendations for diagnosis‐the Porto criteria. J. Pediatr. Gastroenterol. Nutr. 41:1–7. 15990620 10.1097/01.mpg.0000163736.30261.82

[31] Cersini, A. , M. C. Martino , I. Martini , G. Rossi , and M. L. Bernardini . 2003 Analysis of virulence and inflammatory potential of Shigella flexneri purine biosynthesis mutants. Infect. Immun. 71:7002–7013. 14638790 10.1128/IAI.71.12.7002-7013.2003 PMC308888

[32] Lorè, N. I. , C. Cigana , C. Riva , I. De Fino , A. Nonis , L. Spagnuolo , B. Sipione , L. Cariani , D. Girelli , G. Rossi , et al. 2016 IL‐17A impairs host tolerance during airway chronic infection by Pseudomonas aeruginosa. Sci. Rep. 6:25937. 27189736 10.1038/srep25937 PMC4870500

[33] Dixon, M. F. , R. M. Genta , J. H. Yardley , and P. Correa . 1996 Classification and grading of gastritis. The updated Sydney System. International Workshop on the Histopathology of Gastritis, Houston 1994. Am. J. Surg. Pathol. 20:1161–1181. 8827022 10.1097/00000478-199610000-00001

[34] Gavrieli, Y. , Y. Sherman , and S. A. Ben‐Sasson . 1999 Identification of programmed cell death in situ via specific labeling of nuclear DNA fragmentation. J. Cell Biol. 119:493–501. 10.1083/jcb.119.3.493 PMC2289665 1400587

[35] Ciccocioppo, R. , A. Di Sabatino , R. Parroni , P. Muzi , S. D'Alò , T. Ventura , M. A. Pistoia , M. G. Cifone , and G. R. Corazza . 2001 Increased enterocyte apoptosis and Fas‐Fas ligand system in celiac disease. Am. J. Clin. Pathol. 115:494–503. 11293896 10.1309/UV54-BHP3-A66B-0QUD

[36] Levillain, O. , S. Balvay , and S. Peyrol . 2005 Mitochondrial expression of arginase II in male and female rat inner medullary collecting ducts. J. Histochem. Cytochem. 53:533–541. 15805427 10.1369/jhc.4A6489.2005

[37] Harris, L. D. , N. R. Klatt , C. Vinton , J. A. Briant , B. Tabb , K. Ladell , J. D. Estes , D. A. Price , and V. M. Hirsch . 2010 Mechanisms underlying γδ T‐cell subset perturbations in SIV‐infected Asian rhesus macaques. Blood 116:4148–4157. 20660793 10.1182/blood-2010-05-283549 PMC2993620

[38] Favre, D. , S. Lederer , B. Kanwar , Z. M. Ma , S. Proll , Z. Kasakow , J. Mold , L. Swainson , J. D. Barbour , C. R. Baskin , et al. 2009 Critical loss of the balance between Th17 and T regulatory cell populations in pathogenic SIV infection. PLoS Pathog. 5:e1000295. 19214220 10.1371/journal.ppat.1000295 PMC2635016

[39] Brenchley, J. M. , M. Paiardini , K. S. Knox , A. I. Asher , B. Cervasi , T. E. Asher , P. Scheinberg , D. A. Price , C. A. Hage , L. M. Kholi , et al. 2008 Differential Th17 CD4 T‐cell depletion in pathogenic and nonpathogenic lentiviral infections. Blood 112:2826–2835. 18664624 10.1182/blood-2008-05-159301 PMC2556618

[40] Raffatellu, M. , R. L. Santos , D. E. Verhoeven , M. D. George , R. P. Wilson , S. E. Winter , I. Godinez , S. Sankaran , T. A. Paixao , M. A. Gordon , et al. 2008 Simian immunodeficiency virus‐induced mucosal interleukin‐17 deficiency promotes *Salmonella* dissemination from the gut. Nat. Med. 14:421–428. 18376406 10.1038/nm1743 PMC2901863

[41] Manuzak, J. A. , T. Hensley‐McBain , A. S. Zevin , C. Miller , R. Cubas , B. Agricola , J. Gile , L. Richert‐Spuhler , G. Patilea , J. D. Estes , et al. 2016 Enhancement of microbiota in healthy macaques results in beneficial modulation of mucosal and systemic immune function. J. Immunol. 196:2401–2409. 26826246 10.4049/jimmunol.1502470 PMC4761491

[42] Abernethy, N. J. , J. B. Hay , W. G. Kimpton , E. Washington , and R. N. Cahill . 1991 Lymphocyte subset‐specific and tissue‐specific lymphocyte‐endothelial cell recognition mechanisms independently direct the recirculation of lymphocytes from blood to lymph in sheep. Immunology 72:239–245. 2016121 PMC1384490

[43] Veazey, R. S. , M. DeMaria , L. V. Chalifoux , D. E. Shvetz , D. R. Pauley , H. L. Knight , M. Rosenzweig , R. P. Johnson , R. C. Desrosiers , and A. A. Lackner . 1998 Gastrointestinal tract as a major site of CD4+ T cell depletion and viral replication in SIV infection. Science 280:427–443. 9545219 10.1126/science.280.5362.427

[44] Kraal, D. , I. L. Weissman , and E. C. Butcher . 1983 Difference in vivo distribution and homing ofT cell subsets to mucosal vs nonmucosal lymphoid organs. J. Immunol. 130:1097–1118. 6600472

[45] Pals, S. T. , G. Kraal , E. Horst , A. De Groot , R. J. Scheper , and C. J. Meijer . 1986 Human lymphocyte‐high endothelial venule interaction: organ‐selective binding of T and B lymphocyte populations to high endothelium. J. Immunol. 137:760. 3487586

[46] Mehandru, S. , M. A. Poles , K. Tenner‐Racz , P. Jean‐Pierre , V. Manuelli , P. Lopez , A. Shet , A. Low , H. Mohri , D. Boden , et al. 2006 Lack of mucosal immune reconstitution during prolonged treatment of acute and early HIV‐1 infection. PLoS Med. 3:e484. 17147468 10.1371/journal.pmed.0030484 PMC1762085

[47] Mavigner, M. , M. Cazabat , M. Dubois , F.‐E. L'Faqihi , M. Requena , C. Pasquier , P. Klopp , J. Amar , L. Alric , K. Barange , et al. 2012 Altered CD4+ T cell homing to the gut impairs mucosal immune reconstitution in treated HIV‐infected individuals. J. Clin. Invest. 122:62–69. 22156200 10.1172/JCI59011 PMC3248296

[48] Nigam, P. , S. Kwa , V. Velu , and R. R. Amara . 2011 Loss of IL‐17‐producing CD8 T cells during late chronic stage of pathogenic simian immunodeficiency virus infection. J. Immunol. 186:745–753. 21148794 10.4049/jimmunol.1002807

[49] Xu, H. , X. Wang , D. X. Liu , T. Moroney‐Rasmussen , A. A. Lackner , and R. S. Veazey . 2012 IL‐17‐producing innate lymphoid cells are restricted to mucosal tissues and are depleted in SIV‐infected macaques. Mucosal Immunol. 5:658–669. 22669579 10.1038/mi.2012.39 PMC3702374

[50] Cello, J. P. , and L. W. Day . 2009 Idiopathic AIDS enteropathy and treatment of gastrointestinal opportunistic pathogens. Gastroenterology 136:1952–1965. 19457421 10.1053/j.gastro.2008.12.073 PMC7094677

[51] Kamat, A. , P. Ancuta , R. S. Blumberg , and D. Gabuzda . 2010 Serological markers for inflammatory bowel disease in AIDS patients with evidence of microbial translocation. PLoS ONE 5:e15533. 21125014 10.1371/journal.pone.0015533 PMC2981579

[52] Pellecchia, P. , and L. J. Brandt ., 2010 Intestinal abnormalities in AIDS Pp. 753–65 *in* Classen M. Tytgat G. N. J. andLightdale C. J., eds. Gastroenterological endoscopy. Thieme, Stuttgart, Germany.

[53] Ullrich, R. , M. Zeitz , W. Heise , M. L'age , G. Höffken , and E. O. Riecken . 1989 Small intestinal structure and function in patients infected with human immunodeficiency virus (HIV): evidence for HIV‐induced enteropathy. Ann. Intern. Med. 111:15–21. 2500046 10.7326/0003-4819-111-1-15

[54] Mattapallil, J. J. , Z. Smit‐McBride , M. McChesney , and S. Dandekar . 1998 Intestinal intraepithelial lymphocytes are primed for gamma interferon and MIP‐1beta expression and display antiviral cytotoxic activity despite severe CD4(+) T‐cell depletion in primary simian immunodeficiency virus infection. J. Virol. 72:6421–6429. 9658083 10.1128/jvi.72.8.6421-6429.1998 PMC109797

[55] Lundqvist, C. , V. Baranov , K. Söderström , L. Athlin , R. Kiessling , S. Hammarström , and M. L. Hammarström . 1995 Phenotype and cytokine profile of intraepithelial lymphocytes in human small and large intestine. Ann. N. Y. Acad. Sci. 756:395–399. 7645856 10.1111/j.1749-6632.1995.tb44544.x

[56] Targan, S. R. , S. B. Hanauer , S. J. van Deventer , L. Mayer , D. H. Present , T. Braakman , K. L. DeWoody , T. F. Schaible , and P. J. Rutgeerts . 1997 A short‐term study of chimeric monoclonal antibody cA2 to tumor necrosis factor alpha for Crohn's disease. Crohn's Disease cA2 Study Group. N. Engl. J. Med. 337:1029–1035. 9321530 10.1056/NEJM199710093371502

[57] Mogilner, J. G. , I. Srugo , M. Lurie , R. Shaoul , A. G. Coran , E. Shiloni , and I. Sukhotniket . 2007 Effect of probiotics on intestinal regrowth and bacterial translocation after massive small bowel resection in a rat. J. Pediatr. Surg. 42:1365–1371. 17706498 10.1016/j.jpedsurg.2007.03.035

[58] Buccigrossi, V. , G. Laudiero , E. Nicastro , E. Miele , F. Esposito , and A. Guarino . 2011 The HIV‐1 transactivator factor (Tat) induces enterocyte apoptosis through a redox‐mediated mechanism. PLoS ONE 6:e29436. 22216281 10.1371/journal.pone.0029436 PMC3246489

[59] D'Souza, A. , L. Fordjour , A. Ahmad , C. Cai , D. Kumar , G. Valencia , J. V. Aranda , and K. D. Beharry . 2010 Effects of probiotics, prebiotics, and synbiotics on messenger RNA expression of caveolin‐1, NOS, and genes regulating oxidative stress in the terminal ileum of formula‐fed neonatal rats. Pediatr. Res. 67:526–531. 20101198 10.1203/PDR.0b013e3181d4ff2b

[60] Chandra, D. , G. Choy , and D. G. Tang . 2007 Cytosolic accumulation of HSP60 during apoptosis with or without apparent mitochondrial release: evidence that its pro‐apoptotic or pro‐survival functions involve differential interactions with caspase‐3. J. Biol. Chem. 282:31289–31301. 17823127 10.1074/jbc.M702777200

[61] Möbius, J. , S. Groos , A. Meinhardt , and J. Seitz . 1997 Differential distribution of the mitochondrial heat‐shock protein 60 in rat gastrointestinal tract. Cell Tissue Res. 287:343–350. 8995205 10.1007/s004410050759

[62] Tomasello, G. , V. Rodolico , M. Zerilli , A. Martorana , F. Bucchieri , A. Pitruzzella , A. Marino Gammazza , S. David , F. Rappa , G. Zummo , et al. 2011 Changes in immunohistochemical levels and subcellular localization after therapy and correlation and colocalization with CD68 suggest a pathogenetic role of Hsp60 in ulcerative colitis. Appl. Immunohistochem. Mol. Morphol. 19:552–561. 21441812 10.1097/PAI.0b013e3182118e5f

