# Supplementary figures and images for: Probiotic supplementation promotes a reduction in T‐cell activation, an increase in Th17 frequencies, and a recovery of intestinal epithelium integrity and mitochondrial morphology in ART‐treated HIV‐1‐positive patients
Source: Immun Inflamm Dis. 2017 Apr 20;5(3):244–60. doi: 10.1002/iid3.160 (PMC5569369; doi:10.1002/iid3.160)

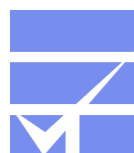

# CONSORT

TRANSPARENT REPORTING of TRIALS

## CONSORT 2010 Flow Diagram

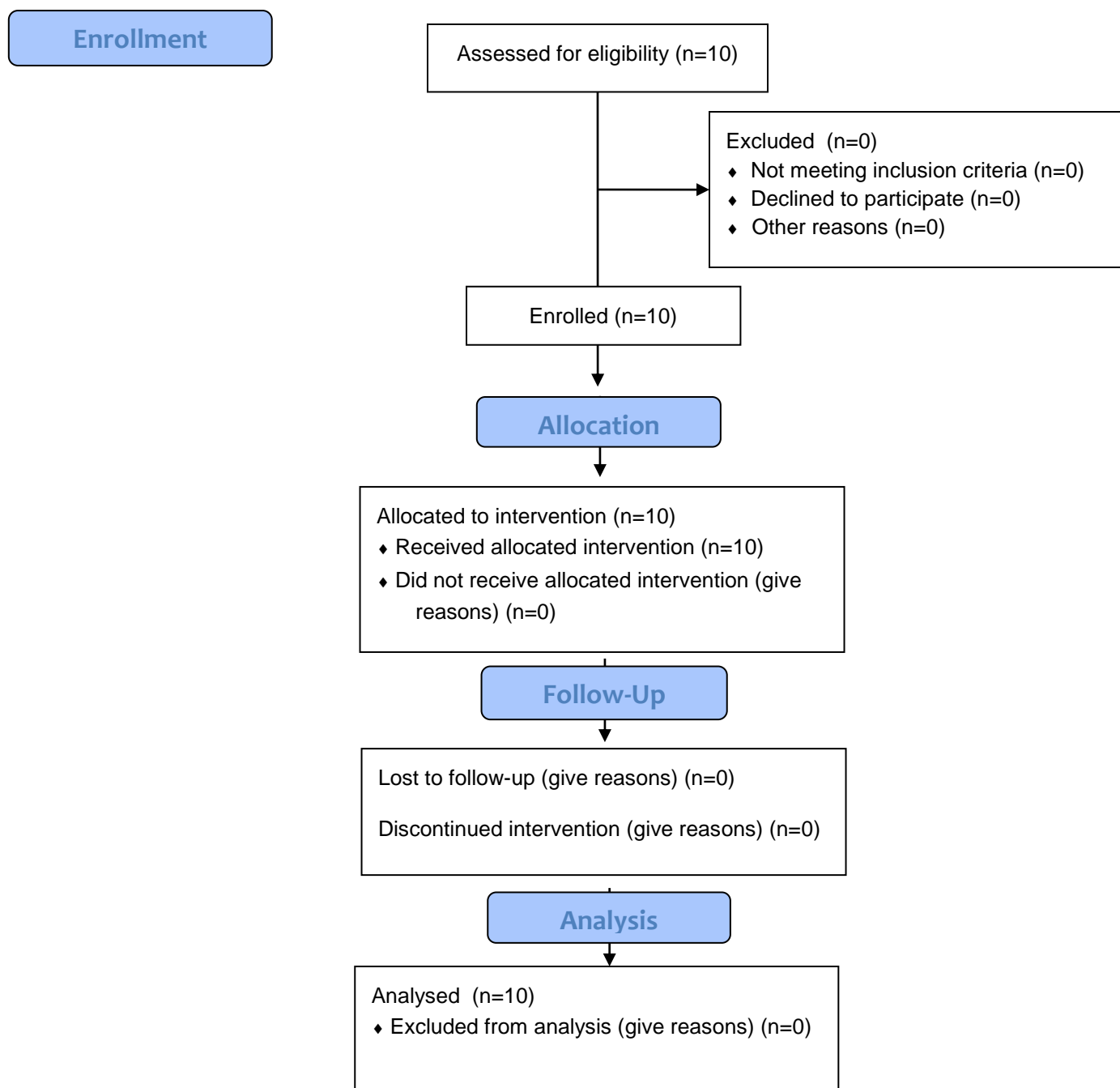

Supplement: Supplementary file 2 — Figure S2. Flow diagram. [file IID3-5-244-s002.pdf]

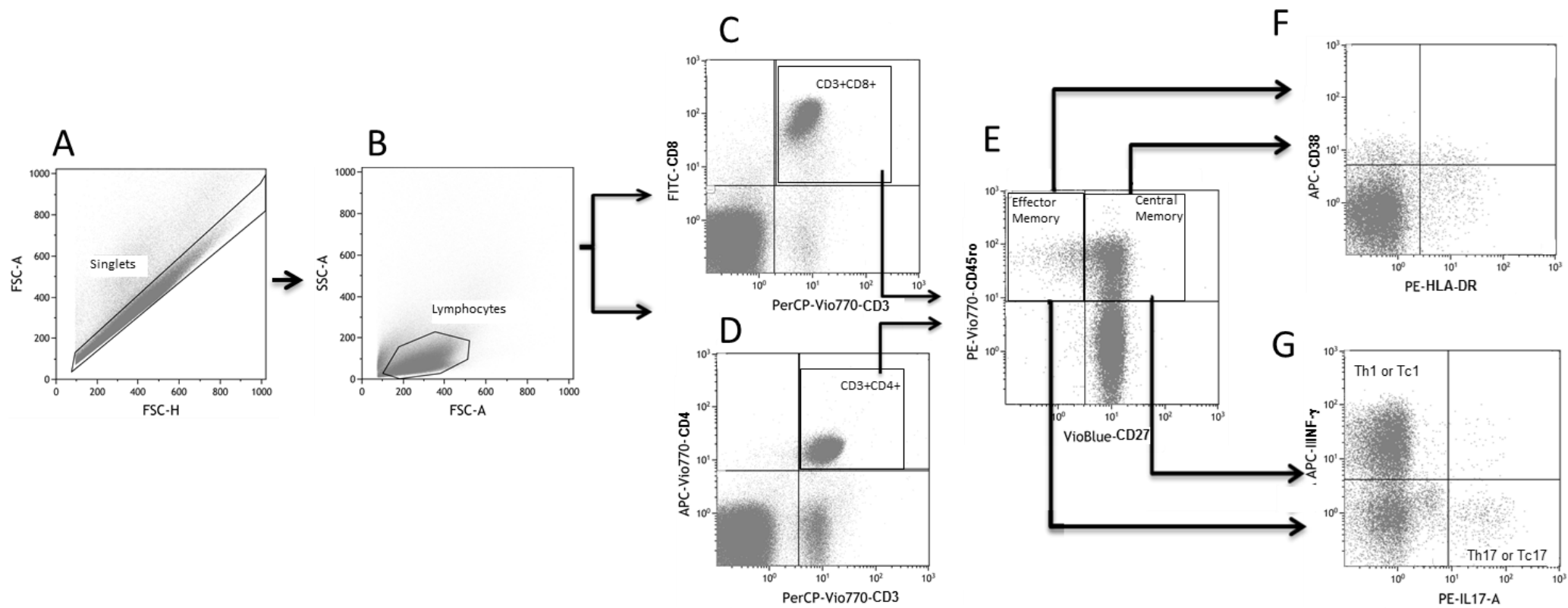

Supplement: Supplementary file 3 — Figure S3. Gating strategy for flow cytometry analysis of peripheral blood and GALT. Singlets were identified by FSC‐A versus FSC‐H dot plot (A). After gating on lymphocytes, identified by the FSC and SSC parameters (B), CD3+CD4+ and CD3+CD8+ gates were defined (C and D); for both of them, central memory (CM) and effector memory (EM) gates were identified by the expression of CD45ro and CD27 (E). CD8 and CD4 lymphocytes and the CM and EM subpopulations were investigated for the activation status by the CD38 and HLA‐DR expression (F) and for the intra‐citoplasmatic expression of IFN‐γ (Th1 and Tc1, respectively) and IL‐17A (Th17 and Tc17, respectively) (G). [file IID3-5-244-s003.pdf]
